# Monoclonal antibody therapy demonstrates increased virulence of a lineage VII strain of Lassa virus in nonhuman primates

**DOI:** 10.1080/22221751.2023.2301061

**Published:** 2024-01-02

**Authors:** Courtney Woolsey, Robert W. Cross, Abhishek N. Prasad, Krystle N. Agans, Viktoriya Borisevich, Daniel J. Deer, Natalie S. Dobias, Alyssa C. Fears, Mack B. Harrison, Megan L. Heinrich, Karla A. Fenton, Robert F. Garry, Luis M. Branco, Thomas W. Geisbert

**Affiliations:** a Galveston National Laboratory, University of Texas Medical Branch, Galveston, TX, USA; b Department of Microbiology and Immunology, University of Texas Medical Branch, Galveston, TX, USA; c Zalgen Labs, LLC, Frederick, MD, USA; d Department of Microbiology and Immunology, Tulane University School of Medicine, New Orleans, LA, USA

**Keywords:** Lassa virus, arenavirus, monoclonal antibodies, haemorrhagic fever, nonhuman primates

## Abstract

Lassa virus (LASV) is a World Health Organization (WHO) priority pathogen that causes high morbidity and mortality. Recently, we showed that a combination of three broadly neutralizing human monoclonal antibodies known as Arevirumab-3 (8.9F, 12.1F, 37.2D) based on the lineage IV Josiah strain protected 100% of cynomolgus macaques against heterologous challenge with lineage II and III strains of LASV when therapy was initiated beginning at day 8 after challenge. LASV strains from Benin and Togo represent a new lineage VII that are more genetically diverse from lineage IV than strains from lineages II and III. Here, we tested the ability of Arevirumab-3 to protect macaques against a LASV lineage VII Togo isolate when treatment was administered beginning 8 days after exposure. Unexpectedly, only 40% of treated animals survived challenge. In a subsequent study we showed that Arevirumab-3 protected 100% of macaques from lethal challenge when treatment was initiated 7 days after LASV Togo exposure. Based on our transcriptomics data, successful Arevirumab-3 treatment correlated with diminished neutrophil signatures and the predicted development of T cell responses. As the *in vitro* antiviral activity of Arevirumab-3 against LASV Togo was equivalent to lineage II and III strains, the reduced protection in macaques against Togo likely reflects the faster disease course of LASV Togo in macaques than other strains. This data causes concern regarding the ability of heterologous vaccines and treatments to provide cross protection against lineage VII LASV isolates.

## Introduction

The 2020–2023 COVID-19 pandemic that caused nearly 7 million deaths worldwide [1] highlights the importance of pandemic preparedness for zoonotic viral pathogens that have the potential to spread from endemic areas. *Lassa mammarenavirus* (LASV) is a member of the family *Arenaviridae* and can cause severe and lethal infections in humans and nonhuman primates (NHP) known as Lassa fever (LF) [2,3]. LASV is endemic in West Africa with the multimammate mouse *Mastomys natalensis* serving as the natural reservoir. LASV infection of humans occurs mostly by contact of mucosal surfaces with dust or food from tainted rodent urine or faeces [2]. While human-to-human transmission is uncommon it can occur after contact with infected body fluids [4].

It is thought that 300,000–500,000 people are infected with LASV annually in West Africa; however, recent studies suggest that the number of cases of LF in endemic areas is severely underreported [5]. Studies have projected that as high as 80% of LASV infections are mild or asymptomatic [6]. The overall case fatality rate for LASV infection is 1%–2%; however, mortality increases markedly in hospitalized patients, with a recent multiyear study in Sierra Leone reporting a case fatality rate of 69% [7]. Previous importations of LF from West Africa to other continents by travellers on commercial airlines [8] demonstrate the potential for spread outside of endemic countries. Symptoms of LF including headache, fever, and fatigue that may progress to sore throat, retrosternal chest pain, abdominal pain, conjunctival injection, vomiting, and diarrhoea. In severe cases hypotension, shock, neurological complications, and multiorgan failure can occur. An assortment of sequelae have been reported after recovery with deafness occurring in up to one-third of LF survivors [9].

There are currently no preventive vaccines or post-exposure treatments approved to combat LF. For this reason as well as its pandemic potential, LASV was recently included on the World Health Organization's (WHO) Blueprint List of Priority Pathogens [10] as well as the Coalition for Epidemic Preparedness Innovations (CEPI) list of Priority Diseases [11] with CEPI investing in the advanced development of several different vaccine candidates. Notably, the WHO recently published a Target Product Profile (TPP) for LF vaccines [12]. Among the desired characteristics is the ability of a vaccine to cross-protect against several different LASV lineages [13]. By natural extension this same TPP also applies to post-exposure treatments and therapies.

Sequence analysis of LASV isolates from endemic areas in West Africa show a unusually high level of genetic diversity, with at least four lineages of LASV defined that associate with geographical regions [14]. LASV lineages I, II, and III are found in Nigeria and appear to be ancestral to lineage IV isolates that are localized in or near Sierra Leona, Liberia, and Guinea. Four additional lineages of LASV have been proposed with viruses from Mali and Côte d’Ivoire, representing a fifth potential lineage [15]; isolates from *Hylomyscus pamfi* rodents in Nigeria making up a sixth lineage [16]; and viruses from Togo [17,18] and Benin [18,19] representing seventh and eighth lineages. The genetic heterogeneity within LASV raises important questions about the efficacy of medical countermeasures that can confer protect efficacy against all LASV lineages (see Supplementary Figure 1 for a comparison of amino acid heterogeneity among several LASV isolates). Preclinical studies in NHPs have shown the potential for two candidate vaccines based on the lineage IV prototype Josiah strain of LASV to cross-protect against heterologous lineages [20–22]. While these initial studies are encouraging, whether these vaccines can protect against strains representative of all LASV lineages is unknown.

In regard to post-exposure treatments and therapies, the most promising preclinical studies in NHPs are based on broadly neutralizing human monoclonal antibodies (BNhuMAbs). Like preventive vaccines these BNhuMAbs have been developed utilizing PBMC from survivors of lineage IV LASV strains [23–25]. We recently showed that a mixture of these BNhuMAbs known as Arevirumab-3 can not only protect NHPs against homologous lineage IV LASV challenge when administered as a therapy initiated at an advanced stage of disease [25], but can also provide heterologous protection of NHPs under identical test conditions against heterologous lineage II and III strains of LASV [26]. While this antibody therapy data is encouraging lineage II and III strains are genetically more closely related than more recently discovered isolates from different lineages. Specifically, the recently emerged and proposed LASV lineage VII has shown an unusually high case fatality rate of 50% in Togo and Benin and this lineage has the highest sequence diversity from lineage IV strains of LASV [19]. Here, we developed a new uniformly lethal cynomolgus monkey model for a lineage VII strain of LASV from Togo and employed this model to determine whether Arevirumab-3 could also provide heterologous protection against this more genetically diverse Togo isolate.

## Results

### NHP model of Lassa lineage VII Togo strain

**Figure 1.**
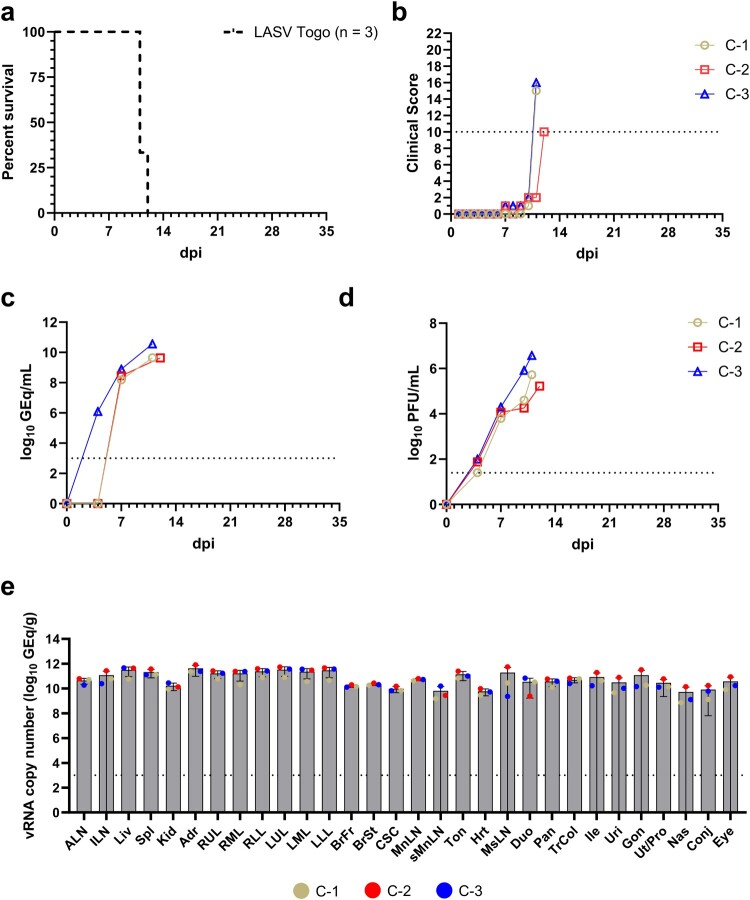
Survival, clinical scoring, virus replication kinetics, and tissue viral load in macaques challenged with LASV lineage VII strain Togo. (a) Kaplan–Meier survival curve for LASV-challenged macaques. (b) Clinical scoring for macaques challenged with LASV. The dashed line denotes the minimum clinical score by which humane euthanasia criteria was met. Viral load was determined by RT-qPCR of LASV genomic RNA (vRNA) from whole blood (c) or plaque titration of infectious virus from plasma (d) collected at pre-determined timepoints. For both panels, individual data points represent the mean of two technical replicates. To fit on a log scale axis, zero values (below the limit of quantitation [LOQ]) are plotted as “1” (10^0^). (e) Tissue LASV vRNA load from macaques challenged with LASV. For (c, d, e), dashed horizontal lines indicate the LOQ for the assay (1000 GEq/mL or GEq/g for RT-qPCR; 25 PFU/mL for plaque titration). To fit on a log scale axis, zero values (below LOQ) are plotted as “1” (10^0^). For (e), bars represent the mean for the cohort, and symbols within bars represent the mean of two technical replicate assays for each individual animal. Error bars represent the upper SD. For all relevant panels, reported *p*-values are two-tailed. Abbreviations for tissues: ALN: axillary lymph node; ILN: inguinal lymph node; Liv: liver; Spl: spleen; Kid: kidney; Adr: adrenal gland; RUL: right upper lung; RML: right middle lung; RLL: right lower lung; LUL: left upper lung; LML: left middle lung; LLL: left lower lung; BrFr: brain frontal cortex; BrSt: brain stem; CSC: cervical spinal cord; MnLN: mandibular lymph node; sMnLN: submandibular lymph node; Ton: tonsil; Hrt: heart; MsLN: mesenteric lymph node; Duo: duodenum; Pan: pancreas; TrCol: transverse colon; Ile: ileum; Uri: urinary bladder; Gon: gonad; Nas: nasal mucosa; Ut/Pro: uterus/prostate; Conj: conjunctiva.

To assess the pathogenic potential of a recent lineage VII LASV isolate we challenged three cynomolgus macaques by intramuscular (i.m.) injection with 1,000 PFU of strain Germany ex Togo/2016/7082 (LASV Togo). All three macaques (C-1, C-2, C-3) showed disease features including anorexia, lethargy, central nervous system anomalies, leukopoenia, thrombocytopenia, hypoalbuminemia, and elevations in circulating levels of liver-associated enzymes and C-reactive protein consistent with previous LF studies [3,22,25,26] (Supplementary Table 1). All three animals reached clinical scores requiring euthanasia on days 11, 11, and 12 post-infection (p.i.), respectively (Figure 1(a,b)). For the LASV Togo-infected macaques circulating viremia levels up to 10.56 log10 genome equivalents (GEq)/ml and 6.57 log10 plaque forming units (PFU)/ml were observed (Figure 1(c,d)) while tissue loads up to 11.74 log10 GEq/g were noted (Figure 1(e)).

### *In vitro* efficacy of Arevirumab-3 against LASV lineage VII strain Togo

Multiple alignment of the glycoprotein complex of several representative isolates from different lineages of LASV demonstrate significant homology in the established binding regions of Arevirumab-3 (Supplementary Figure 1) suggesting that potent virus neutralizing capacity against the Togo isolate may be possible. *In vitro* neutralizing activities of anti-LASV BNhuMAbs individually as well as combined in the Arevirumab-3 cocktail were determined using 50% plaque reduction neutralization tests (PRNT_50_) against the LASV lineage VII isolate Togo (Supplementary Figure 2). For all individual mAbs, 50% neutralization compared to the control plate was achieved at antibody concentrations ∼ 0.39 µg/mL (8.9F), ∼0.8 µg/mL (12.1F), and 20 µg/mL (37.2D) (Supplementary Figure 2(a–c)). As expected, the combined Arevirumab-3 formulation exhibited superior neutralizing activity compared to the individual BNhuMAbs against the Togo isolate (Supplementary Figure 2(d)). The components of Arevirumab-3 were derived from survivors from Sierra Leone where lineage IV LASV strains predominate. Nonetheless, the components of Arevirumab-3, individually and combined, demonstrated comparable neutralization capacity against LASV Togo.

### Protective efficacy of Arevirumab-3 against LASV lineage VII strain Togo

**Figure 2.**
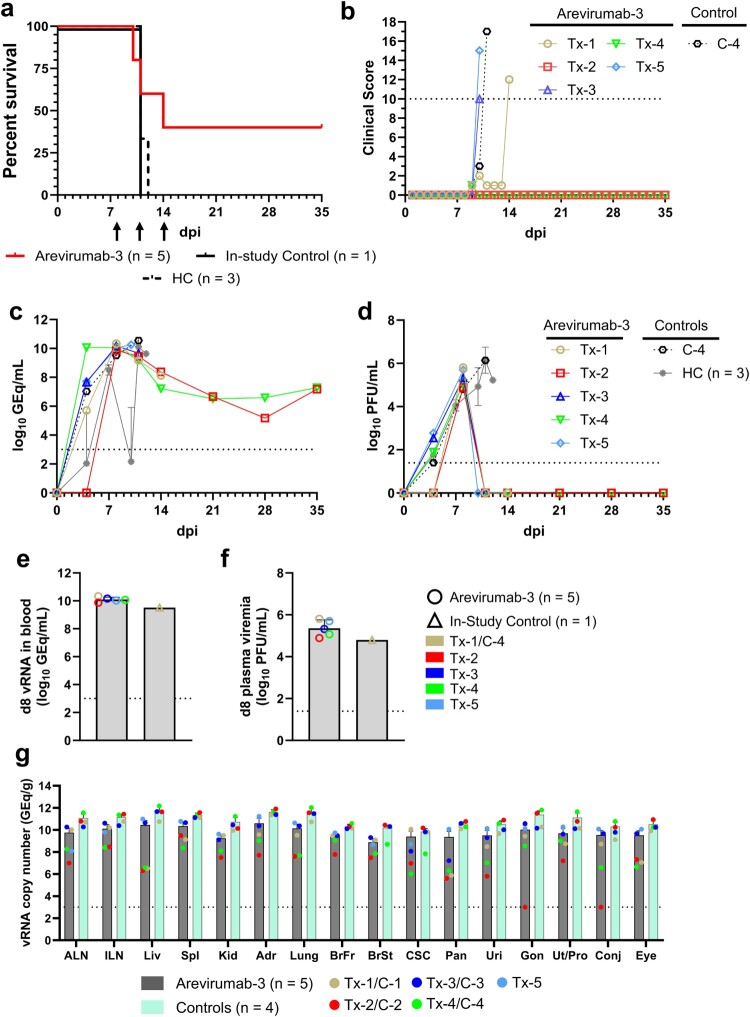
Survival analysis, clinical scoring, virus replication kinetics, and tissue viral load in macaques challenged with LASV lineage VII strain Togo and treated with Arevirumab-3 on 8, 11, and 14 days post-infection. (a) Kaplan–Meier survival curves for LASV-challenged macaques. The curve for the in-study untreated controls animal (C-4) is shown separately from the curve for the additional identically challenged controls from the initial model development study (*n* = 3); however, for statistical comparison all untreated controls were grouped together (*n* = 4). Differences in curves were tested by the Mantel–Cox log-rank test. Arrows below the *x*-axis denote Arevirumab-3 treatment days (days 8, 11, 14). (b) Clinical scoring for macaques challenged with LASV. The dashed line denotes the minimum clinical score by which humane euthanasia criteria was met. (c–f) Viral load was determined by RT-qPCR of LASV vRNA from whole blood (c, e) or plaque titration of plasma (d, f) collected at pre-determined timepoints. (c, d) LASV replication kinetics in macaques as measured by RT-qPCR of LASV vRNA isolated from whole blood (c), or plaque titration of infectious virus from plasma (d). (e, f) Viral load as measured by RT-qPCR of LASV vRNA isolated from whole blood (e) or plaque titration of infectious virus from plasma (f) at the time treatment with Arevirumab-3 was initiated (8 days p.i.). Since untreated animals from the initial model study (Figure 1) were not sampled 8 days p.i., comparisons are to the single in-study control, therefore no statistical comparison was performed. For (c–f), individual data points represent the mean of two technical replicates, except for grouped untreated controls in (c, d), which represent the geometric mean for the cohort ± geometric SD. Dashed horizontal lines indicate the limit of quantitation (LOQ) for the assay (1000 GEq/mL for RT-qPCR; 25 PFU/mL for plaque titration). To fit on a log scale axis, zero values (below LOQ) are plotted as “1” (10^0^). (g) Tissue LASV vRNA load from macaques challenged with LASV and treated with Arevirumab-3. Values below the LOQ are plotted as “999”. Abbreviations for tissues: ALN: axillary lymph node; ILN: inguinal lymph node; Liv: liver; Spl: spleen; Kid: kidney; Adr: adrenal gland; BrFr: brain frontal cortex; BrSt: brain stem; CSC: cervical spinal cord; Pan: pancreas; Uri: urinary bladder; Gon: gonad; Ut/Pro: uterus/prostate; Conj: conjunctiva. HC = historical controls.

To determine if Arevirumab-3 can be effective as a therapeutic treatment against a heterologous LASV exposure, six cynomolgus macaques were challenged with 1,000 PFU of LASV lineage VII strain Togo by i.m. injection (Figure 2). Arevirumab-3 (8.9F, 12.1F and 37.2D) was administered to five animals (Tx-1–Tx-5) by intravenous (i.v.) injection on days 8, 11, and 14 (15 mg/kg of each MAb) after infection. One experimental positive control animal received no treatment (C-4). All six animals showed evidence of LASV disease prior to treatment as indicated by a constellation of clinical signs that varied among animals and included decreased appetite (6/6), lymphopenia (5/6), monocytopenia (5/6); thrombocytopenia (5/6), and elevated circulating levels of AST (6/6) and C-reactive protein (6/6) (Supplementary Table 2). In addition, 6/6 animals were viremic on day 8 p.i. prior to treatment with circulating viral loads ranging from 9.51 to 10.34 log10 GEq/ml and 4.80–5.81 log10 PFU/ml (Figure 2(c–f)). In contrast to previous studies showing that Arevirumab-3 completely protected macaques against heterologous challenge with lineage II and III strains of LASV [26], only 2/5 animals challenged with LASV Togo and treated with Arevirumab-3 survived (Figure 2(a)). The three Arevirumab-3-treated macaques reached clinical scores requiring euthanasia on post-exposure days 10, 11, and 14, respectively, while the positive control animal succumbed on day 11 after infection (Figure 2(a,b)). The two Arevirumab-3-treated animals that survived continued to show clinical signs of illness after LASV challenge particularly perturbations in clinical pathology analytes but mostly returned to baseline values by the day 35 study endpoint when the animals appeared to be healthy with no overt signs of illness.

Compared to earlier timepoints during the acute phase of disease and prior to treatment initiation, relatively low levels of circulating viral genomic RNA (∼7.23 log10 GEq/mL) were still present at the day 35 p.i. study endpoint in both Arevirumab-3-treated macaques that survived (Figure 2(c)) while infectious LASV was cleared by day 11 p.i. in these two animals (Figure 2(d)). Interestingly, in the three animals which developed lethal LF, while circulating viral genomic RNA loads ranged from 8.13 to 10.26 log10 GEq/ml at the time of humane endpoint euthanasia, no circulating infectious LASV was detected by plaque assay in any of these three animals at the same time points. This is likely a result of interference caused by circulating Arevirumab-3 with the plaque assay as high LASV loads were also detected in tissues of these three animals by PCR and immunohistochemistry (IHC) (see below). There was no significant difference in the peak load of both circulating LASV RNA in the Arevirumab-3-treated cohorts compared to the untreated positive control cohort (Supplementary Figure 3(a,b)); however, there was a significant difference in the day both peak vRNA and infectious virus loads were detected (*p* = 0.016 and *p* = 0.008, respectively; Mann–Whitney *U*-test). Tissue viral loads up to 12.18 log10 GEq/g were detected in the experimental positive control macaque and up to 11.23 log10 GEq/g in the three non-surviving Arevirumab-3-treated animals while levels of LASV RNA were lower or undetectable in tissues of the two Arevirumab-3-treated animals that survived to the pre-determined day 35 p.i. study endpoint (Figure 2(g)).

**Figure 3.**
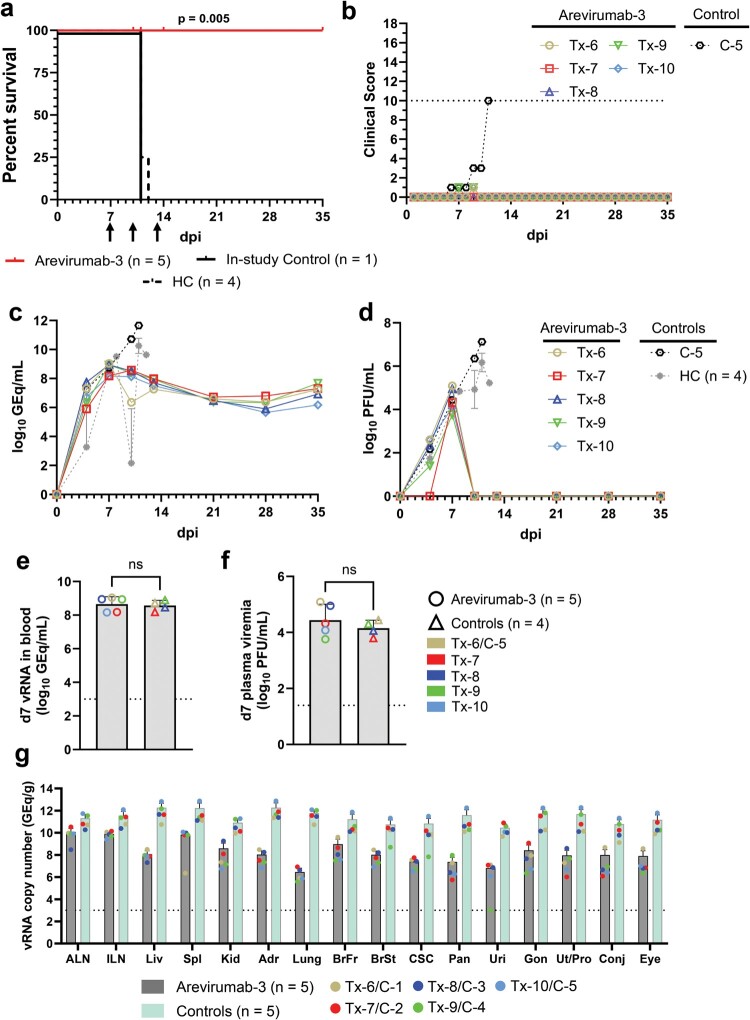
Survival analysis, clinical scoring, virus replication kinetics, and tissue viral load in macaques challenged with LASV lineage VII strain Togo and treated with Arevirumab-3 on 7, 10, and 13 days post-infection. (a) Kaplan–Meier survival curves for LASV-challenged macaques. The curve for the in-study untreated controls animal (C-5) is shown separately from the curve for the additional identically challenged controls from the initial model development study (Figure 1, *n* = 3) and the initial Arevirumab-3 study (Figure 2, *n* = 1); however, for statistical comparison all untreated controls were grouped together (*n* = 5). Differences in curves were tested by the Mantel–Cox log-rank test. Arrows below the *x*-axis indicate Arevirumab-3 treatment days (days 7, 10, 13). (b) Clinical scoring for macaques challenged with LASV. The dashed line denotes the minimum clinical score by which humane euthanasia criteria was met. (c–f) Viral load was determined by RT-qPCR of LASV vRNA from whole blood (c, e) or plaque titration of infectious virus from plasma (d, f) collected at pre-determined timepoints. (c, d) LASV replication kinetics in macaques as measured by RT-qPCR of LASV vRNA isolated from whole blood (c), or plaque titration of infectious virus from plasma (d). (e, f) Viral load as measured by RT-qPCR of LASV vRNA isolated from whole blood (e) or plaque titration of infectious virus from plasma (f) at the time treatment with Arevirumab-3 was initiated (7 days p.i.). Since untreated animals from the initial model study (Figure 1) were also sampled 7 days p.i., these three animals were grouped with the in-study control animal for a total *n* = 4. Statistical comparison was made using the non-parametric Mann–Whitney *U*-test. For (c–f), individual data points represent the mean of two technical replicates, except for grouped untreated controls in (c, d), which represent the geometric mean for the cohort ± geometric SD. Dashed horizontal lines indicate the limit of quantitation (LOQ) for the assay (1000 GEq/mL for RT-qPCR; 25 PFU/mL for plaque titration). To fit on a log scale axis, zero values (below LOQ) are plotted as “1” (10^0^). (g) Tissue LASV vRNA load from macaques challenged with LASV and treated with Arevirumab-3. Values below the LOQ are plotted as “999”. Abbreviations for tissues: ALN: axillary lymph node; ILN: inguinal lymph node; Liv: liver; Spl: spleen; Kid: kidney; Adr: adrenal gland; BrFr: brain frontal cortex; BrSt: brain stem; CSC: cervical spinal cord; Pan: pancreas; Uri: urinary bladder; Gon: gonad; Ut/Pro: uterus/prostate; Conj: conjunctiva. HC = historical controls.

As Arevirumab-3 did not perform as well against heterologous challenge with LASV lineage VII as we had previously shown under identical test conditions when initiated beginning at day 8 p.i. against lineage II or III strains [26], we performed an additional study to see if shortening the therapeutic window between challenge and treatment by 1 day could improve outcome. In this study, six cynomolgus macaques were challenged with 1,000 PFU of LASV lineage VII strain Togo by i.m. injection (Figure 3). Arevirumab-3 (8.9F, 12.1F and 37.2D) was administered to five animals (Tx-6–Tx-10) by i.v. injection on days 7, 10, and 13 (15 mg/kg of each MAb) after infection. One experimental positive control animal received no treatment (C-5). All six animals showed evidence of LASV disease prior to treatment as indicated by a constellation of clinical signs that varied among animals and included decreased appetite (6/6), leukopoenia (6/6); lymphopenia (6/6), monocytopenia (4/6); neutropenia (6/6), thrombocytopenia (6/6), and elevated circulating levels of AST (6/6) and C-reactive protein (4/6) (Supplementary Table 3). In addition, 6/6 animals were viremic on day 7 p.i. prior to treatment with circulating viral loads ranging from 8.18 to 9.04 log10 GEq/ml and 3.76–5.09 log10 PFU/ml (Figure 3(c–f)). Importantly, all animals challenged with LASV Togo and treated with Arevirumab-3 survived with minimal to no clinical scoring after treatment (Figure 3(b)), whereas the untreated positive control animal reached a clinical score requiring euthanasia on day 11 (Figure 3(a,b)). There was a significant difference in the survival curves between the Arevirumab-3-treated cohort and the pooled untreated positive controls (Figure 3(a), *p* = 0.005, Mantel–Cox log-rank test).

As in the previous study where Arevirumab-3 treatment was initiated at 8 dpi, relatively low levels of circulating viral genomic RNA (6.17–7.68 log10 GEq/mL) were still present at the day 35 p.i. study endpoint in all five of the Arevirumab-3-treated macaques that survived (Figure 3(c)), while infectious LASV was cleared by day 10 p.i. in these all Arevirumab-3-treated animals (Figure 3(d)). There was a significant difference in both the peak load of circulating LASV RNA in the Arevirumab-3-treated cohort compared to the untreated positive control cohort (Supplementary Figure 4(a), *p* = 0.004, Mann–Whitney *U*-test), and peak levels of circulating infectious virus in animals treated with Arevirumab-3 compared to untreated controls (Supplementary Figure 4(b), *p* = 0.008, Mann–Whitney *U*-test), as well as the day in which each measure was detected (*p* = 0.008 for both comparisons). Tissue viral loads up to 12.88 log10 GEq/g were detected in the experimental positive control macaque while levels of LASV RNA were lower or undetectable in tissues of the Arevirumab-3-treated animals that all survived to the pre-determined day 35 p.i. study endpoint (Figure 3(g)).

### Gross lesions and histopathology

**Figure 4.**
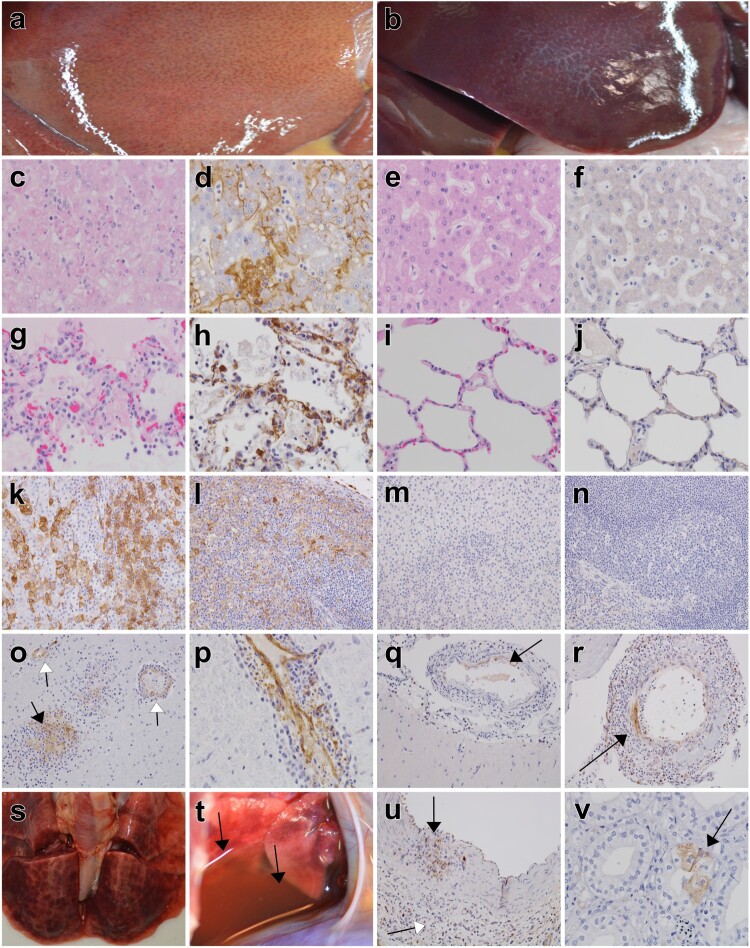
Representative gross, histopathology, and immunohistochemistry for anti-LASV antigen of macaques challenged with LASV Togo. (a) necrotizing hepatitis (C-3); (b) no appreciable gross lesions of the liver (Tx-8); (c) H&E, liver, 40× necrotizing hepatitis (C-3); (d) IHC (brown), 40× liver, LASV antigen positive hepatocytes, mononuclear cells within sinusoids, and sinusoidal lining cells (C-3); (e) H&E, 40× liver, no significant lesions (Tx-6); (f) IHC (brown), 40× liver, no significant immunolabelling (Tx-6); (g) H&E, 40× lung, interstitial pneumonia with oedema, haemorrhage and fibrin accumulation within alveolar spaces (C-3); (h) IHC (brown), 40× lung, LASV antigen positive alveolar macrophages, alveolar septal macrophages, and endothelium (C-3); (i) H&E, 40× lung, no significant lesions (Tx-8); (j) IHC (brown), 40× lung, no significant immunolabelling (Tx-8); (k) IHC (brown), 20× adrenal gland, LASV antigen positive adrenal cortical cells (C-3); (l) IHC (brown), 20× spleen, LASV antigen positive mononuclear cells within the red pulp, white pulp and peritoneal mesothelium (C-3); (m) IHC (brown), 20× adrenal gland, no significant immunolabelling (Tx-6); (n) IHC (brown), 20× spleen, no significant immunolabelling (Tx-6); (o) IHC (brown), 20× brain, LASV antigen positive glial nodule (black arrow) and endothelium (white arrow) (C-2); (p) IHC (brown), 40× brainstem, LASV antigen positive endothelium with perivascular cuffing (C-2); (q) IHC (brown), 20× brain, LASV antigen positive smooth muscle of inflamed meningeal vessel (arrow) (Tx-4); (r) IHC (brown), 20× brainstem, LASV antigen positive smooth muscle (arrow) and mononuclear cells surrounding and infiltrating a meningeal vessel (Tx-7); (s) haemorrhagic interstitial pneumonia (C-3); (t) pleural effusion (black arrows) (C-3); (u) IHC (brown), 20×, kidney, IHC positive infiltrating mononuclear cells within the smooth muscle of a large calibre artery (black arrow) and perivascular inflitrates (Tx-2); (v) IHC (brown), 40×, kidney, IHC positive collecting duct epithelium (arrow) (Tx-8).

No appreciable gross lesions were noted in any of the seven Arevirumab-3-treated macaques that survived LASV challenge (Tx-2, Tx-4, Tx-6, Tx-7, Tx-8, Tx-9, and Tx-10). All three LASV-infected animals from the initial study to establish a model for LASV Togo (C-1–C-3), the two in-study positive control macaques (C-4, C-5), and three Arevirumab-3-treated animals that succumbed to LF (Tx-1, Tx-3, Tx-5) had one or more of the following gross lesions: necrotizing hepatitis (Figure 4(a)), splenomegaly, lymphadenomegaly, haemorrhagic interstitial pneumonia (Figure 4(s)), haemorrhagic adrenalitis, pleural effusion (Figure 4(t)), ascites, pericardial effusion, and congestion of the meninges.

Multiple tissues representing the major target organs of LASV (liver, spleen, adrenal gland, kidney, and lung) and immune privileged sites (brain, reproductive organs, eyes) were examined histologically. All LASV-infected NHPs that succumbed to disease (C-1–C-5, Tx-1, Tx-3, Tx-5) had similar histologic lesions consistent with previous reports of LF [3,20–22,25,26]. The target organs displayed inflammatory and necrotic lesions with colocalized immunolabelling for anti-LASV antigen (Supplementary Table 4). Necrotizing hepatitis characterized as multifocal loss of normal hepatic architecture with accumulations of karyorrhectic and cellular debris with lymphoplasmacytic, histiocytic and lesser neutrophilic inflammation was present in the liver (Figure 4(c)). Positive immunolabelling of histiocytes (Kupffer cells), endothelium and occasionally individual to clusters of hepatocytes was apparent (Figure 4(d)). Lymphocytolysis with increased numbers of tingible body macrophages, red pulp expansion with histiocytes were the prominent findings in the spleen. Mononuclear cells within the red and white pulp, endothelium of associated vessels throughout the examined tissue, and occasionally the peritoneal mesothelium was LASV-antigen positive by IHC (Figure 4(l)). Congestion with a slight influx of mononuclear cells were prominent in the adrenal gland. Immunolabelling of histiocytes, endothelium, and clusters of cells within all layers of the cortex (zona glomerulosa, zona fasciculata and zona reticularis) and medulla was noted (Figure 4(k)). Renal lesions consisted of lymphoplasmacytic perivasculitis and pyelonephritis with LASV-positive cells in the endothelium, glomerular tufts, smooth muscle arteries, and rarely tubular epithelium.

Pulmonary lesions in all LASV Togo-infected macaques that succumbed to disease consisted of mixed lymphoplasmacytic and neutrophilic inflammation expanding the pulmonary alveolar septa and flooding of alveolar spaces with oedema, fibrin, increased numbers of alveolar macrophages, and occasionally haemorrhage (Figure 4(g)). Endothelium and alveolar macrophages were also positive for LASV antigen (Figure 4(h)). In the brain, multifocal lymphohistiocytic perivascular cuffing with occasional vasculitis and glial nodule formation were present. Immunolabelling of the endothelium of vessels, epithelium of the choroid plexus, and glial nodules typify the IHC labelling (Figure 4(o,p)). Mild lymphoplasmacytic infiltrates were prominent in the prostate. Endothelium was immunopositive for LASV antigen in association with the inflammation. Minimal influx of mononuclear cells was noted in the ovary and uterus. Endothelium, mononuclear cells, and thecal cells in the ovary were immunopositive for LASV antigen. Vasocentric lymphohistiocytic inflammation expanded the epididymal stroma and minimally infiltrated the interstitial spaces in the testis. Immunolabelling was largely associated with the endothelium of epididymal vessels and scattered histiocytes in the stroma. Rarely tubules within the epididymis were necrotic with IHC-positivity. Mild lymphohistiocytic inflammation that was IHC positive for LASV antigen was noted in the uveal tract of the eye.

Arevirumab-3-treated NHPs that survived LASV challenge lacked histologic changes and immunolabelling in the examined organs, excluding kidney, brain and to a lesser extent gonads (Figure 4(e,f,i,j,m,n)). Lymphohistiocytic perivasculitis and/or vasculitis with IHC positive smooth muscle of arteries, occasionally mononuclear cells, and rarely the renal collecting duct epithelium were observed in survivors (Figure 4(q,r,u,v)).

### Persistence of LASV in tissues

As all seven Arevirumab-3-treated macaques that survived LASV challenge had low levels of LASV RNA in tissues at the day 35 p.i. study endpoint consistent with previous studies [25,26]; we attempted to isolated infectious LASV from target tissues with the highest LASV RNA levels including inguinal lymph node (Tx-6), axillary lymph node (Tx-7), and spleen (Tx-10). We also attempted to isolate infectious LASV from immunoprivileged tissues (eyes, brain, and testis or ovary) of four of the surviving macaques (Tx-4, Tx-6, Tx-7, Tx-7) selected for higher levels of LASV RNA. Importantly, we were unable to detect any infectious LASV in any tissues of these surviving animals.

### Transcriptomics

**Figure 5.**
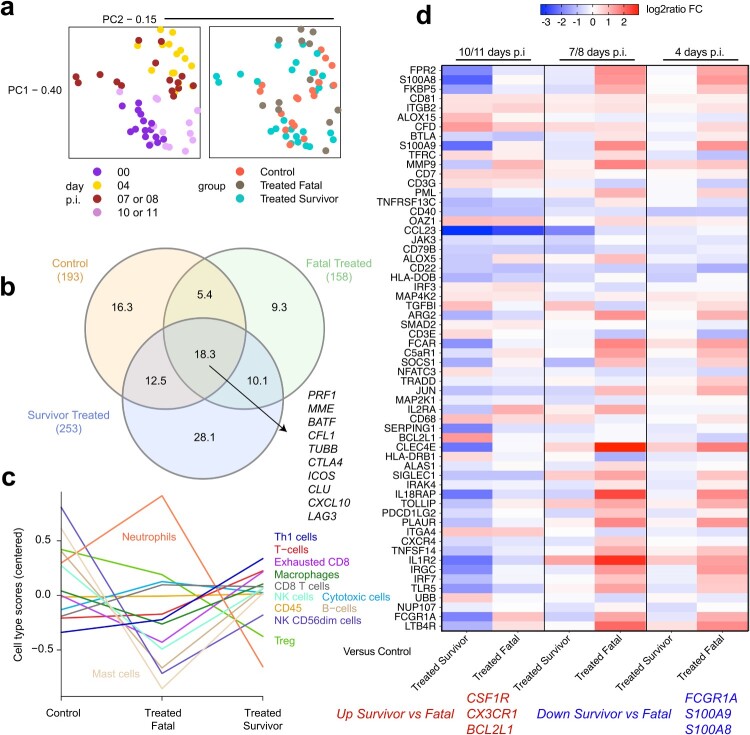
Transcriptional changes in macaques exposed to LASV-Togo and treated with Arevirumab-3 or a vehicle control. (a) Principal component analysis (PCA) of all normalized transcripts to visualize the relatedness of samples via dimensional reduction. Samples were sorted by day post-infection (days p.i.: 0, 4, 7/8, 10/11) and treatment regimen (Control (*n* = 5; C-1, C-2, C-3, C-4, C-5); Treated Fatal (*n* = 3; Tx-1, Tx-3, Tx-5); Treated Survivor (*n* = 7; Tx-2, Tx-4, Tx-6, Tx-7, Tx-8, Tx-9, Tx-10)). Each dot represents an individual RNA sample. (b) Venn diagram depicting overlapping differentially expressed transcripts (Benjamini–Hochberg adjusted *p*-value < 0.05) in control, treated fatal, and survivor groups at 10/11 days p.i. versus a pre-challenge baseline (0 days p.i.). (c) Immune cell type profiling based on transcriptional changes in each group at 10/11 days p.i. (d) Heatmap of the most differentially expressed mRNAs in each treatment group compared to the vehicle control cohort at 10/11, 7/8, or 4 days p.i.

To ascertain immune indicators linked with the protective effects of Arevirumab-3, we conducted targeted transcriptomics on whole blood RNA samples obtained from macaques exposed to LASV Togo. A particular goal was to discover distinct patterns of gene expression linked to successful and failed treatment outcomes. We initiated dimensionality reduction using principal component analysis (PCA) to assess the contribution of two parameters to the overall variability (PC1: 0.40; PC2: 0.15) within the dataset. Specifically, we investigated treatment group (Control (*n* = 5; C-1, C-2, C-3, C-4, C-5), Treated Fatal (*n* = 3; Tx-1, Tx-3, Tx-5), and Treated Survivor (*n* = 7; Tx-2, Tx-4, Tx-6, Tx-7, Tx-8, Tx-9, Tx-10)) and days p.i. at 0, 4, 7/8, 10/11, considering the shared timepoints between these groups. Analysis revealed time-dependent expression changes, but minimal spatial variation was ostensible between groups (Figure 5(a)).

An evaluation of differentially expressed (DE) transcripts (Benjamini–Hochberg false discovery rate (FDR) with a *p*-value below 0.05) for each group versus a pre-challenge baseline (0 days p.i.) revealed distinctive transcriptional profiles for control, treated fatal, and treated survivor groups (Figure 5(b)). In this examination, we focused on the 10/11 days p.i. timepoint because the gene expression profiles of survivors displayed the most significant divergence from both the non-surviving individuals and the control subjects during this timeframe, as indicated by PCA and the DE results (Data S1). Approximately 18.3% of DE mRNAs were shared among all groups, and 16.3%, 9.3%, and 28.1% of transcripts were uniquely expressed in the control, fatal, and survivor groups, respectively. The overlap between the control and fatal, control and survivor, and fatal and survivor, groups constituted 5.4%, 12.5%, and 10.1% of DE transcripts, respectively.

Digital cell quantitation (DCQ) at 10/11 days p.i. was next employed via transcriptional profiling to capture shifts in circulating cell populations (Figure 5(c)). Successful Arevirumab-3 treatment was associated with a predicted increase in frequencies of Th1 cells, T cells, macrophages, and CD8 T cells. Conversely, poor prognosis following Arevirumab-3 treatment was accompanied by a predicted increase in neutrophils and a decrease in mast cells, various T cell subsets, and B cells.

To identify gene expression patterns associated with passive antibody protection, we conducted a comparison of DE transcripts between the positive control cohort versus fatal and survivor groups that received Arevirumab-3 treatment (depicted in Figure 5(d)). Gene expression profiles of treated fatal subjects at the 4 and 7/8 days p.i. timepoints bared a strong resemblance to one another with high expression of transcripts involved in interleukin-1 superfamily (*IL18RAP*, *IL1R2*) and damage-associated molecular pattern (DAMP; *CLEC4E*, *S100A9*, *S100A8*) signalling. Notably, heterodimers of S100A8 and S100A9 form the calcium-binding protein calprotectin, and these serve as a biomarker of lethal LASV [27] and *Bundibugyo ebolavirus* [28] disease. In the treated survivor group, these same mRNAs were strongly downregulated at 10/11 days p.i., indicating their importance in driving a fatal outcome. Higher levels of transcripts encoding T cell (*CD3E*, *NFATC3*) and macrophage (*CD68*) markers were found in the treated survivor group, in line with the DCQ results. The treated survivor group also expressed higher levels of transcripts involved in immunoregulation (*TGFB1*), apoptosis inhibition (*BCL2L1*), antigen presentation (*HLA-DRB1*), and ubiquitination (*UBB*).

## Discussion

A primary concern with developing vaccines and therapies against LASV is the ability of any intervention to provide protection against isolates from genetically diverse lineages. In addition, there appears to be differences among strains within the same lineage in regard to virulence in primates. Recently, we established new NHP models for lineage II (0043/LV/14) and lineage III (Ojoko) LASV strains to assess the cross-protective properties of Arevirumab-3 [26]. Importantly, both isolates caused high lethality and pathogenesis consistent with lineage IV Josiah-like lesions in macaques. The disease caused by 0043/LV/14 and Ojoko in NHPs was also consistent with a lineage VII Lassa isolate from Benin [29]; however, this report utilized a subcutaneous challenge route which may contribute to observed similarities and that a more direct comparison using identical challenge routes and doses may have value. Interestingly, our lineage II 0043/LV/14 and lineage III Ojoko isolates appear to be more pathogenic with clinical signs consistent with those reported for Josiah than other lineage II and III isolates that have been evaluated in macaques [29,30], as well as a lineage V isolate [31] where reduced lethality and atypical disease were reported. Likewise, the lineage VII Togo isolate from the current study appears to exhibit an even faster disease course than our lineage II and lineage III isolates. To illustrate this point, the five untreated Togo-challenged positive control animals from the present study were grouped with two historical control macaques challenged with the identical LASV Togo seed stock and route (*n* = 7, mean time to death [MTD = 10.9 ± 0.6 dpi]) and compared to historical positive control LASV 0043/LV/14- (*n* = 28, MTD = 13.0 ± 2.0 dpi) and Ojoko-infected macaques (*n* = 5, MTD = 14.6 ± 1.4 dpi) [20,26]. A statistically significant difference in the survival curves was observed when compared to either lineage II or lineage III viruses (Supplementary Figure 6; multiplicity-corrected *p* = 0.003, Mantel–Cox log-rank test). Also, the viremia at 8 dpi in macaques infected with LASV Togo appears to be 1–2 log10 GEq/ml higher than at the same time point in our lineage II LASV 0043/LV/14- and lineage III Ojoko-infected monkeys [26] while the 7 dpi viremia with LASV Togo appears to be consistent with the day 8 viremia in our LASV 0043/LV/14- and Ojoko-infected macaques [26].

The current study showing that the contemporary lineage VII Togo isolate is more pathogenic in macaques than strains from several other LASV lineages when using similar challenge approaches and that this difference resulted in a change in efficacy of a medical countermeasure is concerning. This is not unique to LASV. For example, the Angola strain of Marburg virus has been associated with higher case fatality rates in humans and causes a more rapid disease course in NHPs than other Marburg strains [32,33]. Similarly, the Bangladesh strain of Nipah virus causes higher case fatality rates and is more virulent in humans and NHPs than the Malaysia strain [34]. Like our current study, the increased virulence of the Bangladesh strain of Nipah virus in NHPs also resulted in a reduction of efficacy of a human MAb therapy versus the less pathogenic Malaysia strain [34]. The persistence of circulating LASV genomic RNA in surviving animals following Arevirumab-3 treatment, along with an apparent increase in copy number detection between 14 and 35 dpi, supports this notion. Moreover, as the study duration was limited to 35 dpi, it is unknown for how long following Arevirumab-3 treatment LASV genomic RNA may continue to be detected. Persistence of infectious virus in tissues (such as immune privileged “sanctuary” sites) is a major concern with any post-exposure therapeutic, and LASV sequelae in humans may present as sensorineural hearing loss, ophthalmological abnormalities, verbal deficits, alopecia, ataxia, cerebellar ataxia [35]. It is important to note that we were unable to recover infectious LASV from plasma of survivors at any timepoint following Arevirumab-3 treatment, nor from tissues harvested at the study endpoint. Thus, it is likely that some degree of viral replication was allowed to occur following treatment initiation, but that progeny virions were rapidly neutralized. Alternatively, given that the half-life of therapeutic antibodies in NHPs is reported to range widely from 79 to 648 h [36], it is possible that the lack of recovery of infectious LASV from the plasma of surviving animals is due to assay interference by remaining Arevirumab-3, as we postulated was the reason for the lack of detectable virus from treated animals which succumbed to disease in the 8 dpi treatment study. While we cannot rule this out, by the study endpoint (35 dpi), it is likely that both humoral and cell-mediated immune arms have engaged against the infection, helping to limit or eliminate infectious virus. Lastly, given the limited IHC-positivity in some tissues from surviving macaques, and as our study endpoint was limited to 35 dpi, we cannot entirely exclude the possibility of recrudescence and resulting LF sequelae, though at the study endpoint, all surviving macaques were free of clinical signs and markers of disease at latest 12 dpi, and it is possible that the antigen detected in these animals was residual in nature. Indeed, additional verification through staining of different control tissues (e.g. non-infected control, non-specific virus control, secondary antibody-only control), or detection of additional viral proteins (e.g. NP) is necessary to confirm persistence. In addition, future studies will need to address concerns regarding vaccine efficacy against LASV Togo as it will be important to know whether current vaccine candidates based on the lineage IV Josiah strain can protect against LASV Togo.

Our transcriptomics findings support that administering Arevirumab-3 as late passive therapy effectively hinders or reduces LASV disease, enabling the development of an adaptive response. This hypothesis is corroborated by the elevated transcription of molecules associated with antigen presentation and the recruitment of T lymphocytes in macaque subjects that underwent treatment and survived. Instead, an unfavourable treatment result was associated with an anticipated excess of neutrophils, a projected reduction in mast cells and T cells, and increased expression of molecules involved in interleukin-1 and DAMP signalling.

In the context of LASV infection, studies suggests that neutrophils can have both beneficial and detrimental effects. Neutrophils play a role in early antiviral defence but can also contribute to tissue damage if not properly regulated [37]. For example, neutrophils can release various inflammatory mediators that contribute to cytokine storms, which are associated with severe LF and organ damage [27]. While mast cells are typically associated with allergic reactions and inflammation, they also play a role in defence against pathogens [38], potentially including viruses like LASV.

Immunity to LASV appears centred around T cell responses rather than B cell responses [39]. Two main lines of evidence support this. First, T cells (both CD4+ and CD8+) are activated early in infection in human survivors and persist post-recovery, despite limited antibody responses during illness. Second, vaccines inducing T cell responses against LASV glycoproteins, rather than strong antibody responses, provide protection in guinea pigs and NHPs, highlighting T cell responses as crucial determinants of disease defence.

It has been estimated that up to 600 million people could be at risk of developing LF in the future as a result of a number of different environmental factors [40]. Therefore, it is likely that in addition to the currently recognized four lineages of LASV and three or four proposed lineages, new lineages will continue to emerge. While Arevirumab-3 can still provide significant protection when administered to NHPs at an advanced stage of infection (7 dpi), it is not as potent against LASV Togo than it is against strains from three different lineages. Indeed, a multiple alignment of the glycoprotein complex across related clades of LASV demonstrate that homology to the identified binding epitopes for the monoclonal antibody components of Arevirumab-3 are largely conserved (Supplementary Figure 1). Thus, it will be important to continue to optimize mAbs like Arevirumab-3 for broader coverage, as well as to identify other antivirals targeting other aspects of LASV replication that may be combined with mAbs for increased efficacy. Combination strategies have been employed for other viruses to increase efficacy and lengthen the therapeutic window such as we have recently shown in NHP models for Marburg virus [41] and *Sudan ebolavirus* [42]. Specifically, we were able to increase the therapeutic window for treatment by 1 day by combining mAbs with remdesivir. Similar approaches may have utility in treating severe cases of LF.

## Methods summary

Full details of this study's methods are described in the Supplementary Materials. Methods are briefly listed here. A lineage VII LASV isolate Germany ex Togo/2016/7082 originated from serum of a LASV-infected patient in Germany that was exported from Togo [17]. The BNhuMAbs 8.9F, 12.1F, and 37.2D were prepared as previously described [25,26]. *In vitro* plaque reduction neutralization tests (PRNT_50_) were performed as previously described [43]. Fifteen healthy cynomolgus macaques were used to conduct a pilot model study and two separate therapeutic studies. In the first therapeutic treatment study summarized in Supplementary Figure 4 six macaques were challenged with LASV Togo. Arevirumab-3 was administered to 5 macaques 8 days after LASV Togo infection. Additional doses of Arevirumab-3 were given on days 11 and 14 after LASV Togo infection. The virus positive control animal was not treated in this study. Surviving animals were euthanized at the pre-determined study endpoint on day 35 after LASV Togo infection. In the second therapeutic treatment study summarized in Supplementary Figure 4 six macaques ∼ 2.5 were challenged with LASV Togo. Arevirumab-3 was administered to 5 macaques 7 days after LASV Togo infection. Additional doses of Arevirumab-3 were given on days 10 and 13 after LASV Togo infection. The virus positive control animal was not treated in this study. Surviving animals were euthanized at the pre-determined study endpoint on day 35 after LASV Togo infection. All animals for all three studies were given physical examinations, and blood was collected before virus challenge (day 0); and on days 4, 7 or 8, 10 or 11, 13 or 14, 21, 28, and 35 after virus challenge.

Haematology and serum biochemistry was performed as previously described [26]. RNA was isolated from blood of all NHPs before and after LASV Togo infection and from tissues of all animals that succumbed or survived as previously described [26]. LASV Togo RNA was quantified using the CFX96 detection system and the following primer/probe sequences: Forward: 5′ – ACA GTT GCA AAT GGT GTG CT – 3′; Reverse: 5′ – TGG CAG TGA TCT TCC CAT GT – 3′; Probe: 6-carboxyﬂuorescein (FAM)-5 = TGC CTC TCC CAG AGT CAA GTG CA -3 = -6 carboxytetramethylrhodamine (TAMRA). Threshold cycle (CT) values representing viral genomes were analyzed with CFX Manager software, and the data are shown as genome equivalents (GEq). Virus titration was performed by plaque assay using Vero 76 cells (ATCC CRL-1587) from all plasma or tissue samples as previously described [43]. We conducted targeted transcriptomics on macaque blood samples following established protocols [44]. The nCounter .RCC files were imported into NanoString nSolver 4.0 software. To address variations in RNA inputs and reaction efficiency, we utilized a set of 10 housekeeping genes and introduced spiked-in positive and negative controls for raw read count normalization, as previously outlined [45,46].

Necropsy was performed on all subjects. Tissue samples of all major organs were collected for histopathologic and immunohistochemical (IHC) examination, immersion-fixed in 10% neutral buffered formalin, and processed for histopathology, as previously described [26]. Relative severity scores for histological lesions/immunoreactivity were assigned by an ACVP board-certified veterinary pathologist. Representative photomicrographs were qualitatively considered to display lesions that were nominally or ordinally measured by masking of the veterinary pathologist post-examination and ranking lesions to satiate the study objectives, as previously established.

Details about the specific statistical methods used for each comparison are listed in the main text and figure legends. Statistical analysis was performed using Graphpad Prism v10.0.0 and Microsoft Excel. Unless otherwise noted, all reported *p*-values are two-tailed. Statistical significance for DE transcripts was determined by a multiple hypothesis Benjamini–Hochberg FDR-corrected *p*-value less than 0.05.

## Supplementary Material

Data_S1

Supplementary_Figures_23November23

Supplementary_Methods_23November23

Supplementary_Tables

## Data Availability

All study data are included in the main text or the supporting information.
