# Supplementary material for: Monoclonal antibody therapy demonstrates increased virulence of a lineage VII strain of Lassa virus in nonhuman primates
Source: Emerg Microbes Infect. 2024 Jan 2;13(1):2301061. doi: 10.1080/22221751.2023.2301061 (PMC10810630; doi:10.1080/22221751.2023.2301061)
Supplement: Supplementary_Figures_23November23 [file TEMI_A_2301061_SM3663.docx]

**Supplementary Figure 1:** Alignment of LASV lineage II, III, IV, and VII glycoprotein amino acid sequences. The amino acid sequences of Lassa virus glycoprotein gene (GPC) from 0043/LV/14 (LII, Nigeria), Ojoko (LIII, Nigeria), the prototypic Josiah (LIV, Sierra Leone), BEN-16131 (LVII, Benin) and Togo (LVII, Togo) strains were aligned with the Clustal Omega Multiple Sequence Alignment tool (www.ebi.ac.uk/Tools/msa/clustalo/), using default settings. Conservative (:), nonconservative (.), and radical (blank) amino acid replacements, as well as identical residues (*) between the four polypeptides are noted below the alignments. The contact residues for each of the three monoclonal antibodies are colored and bolded: Purple = residues contacted by 8.9F; Green = residues contacted by 12.1F; Dark orange = residues contacted by 37.2D. Contact residues shared by 12.1F and 8.9F are bolded and underlined in dark blue. Critical escape residues thus far identified for each of the monoclonal antibodies are highlighted: 12.1F – bright green; 8.9F – cyan; 37.2D – gray (see also Li et al. Sci Transl Med. 2022 [1]).

**a**

**b**

**c**

**d**

**Supplementary Figure 2: *In vitro* neutralization of LASV lineage VII strain Togo by BNhumAbs.** BNhumAbs targeting the LASV glycoprotein were assessed for plaque reduction neutralization activity against LASV Togo individually **(a-c)** or in combination **(d)** in two-fold serial dilutions. For each panel, the horizontal dashed line indicates 50% neutralization versus the virus control plate. Individual data points represent calculated percent neutralization derived from the mean of duplicate assays.

**b**

**a**

Arevirumab-3 (n = 5)

Tx-1/C-1

Tx-3/C-3

Tx-5

Tx-4/C-4

Controls (n = 4)

Tx-2/C-2

**Supplementary Figure 3:** **Comparison of peak viral load and day of detection in LASV-challenged macaques treated with Arevirumab-3 beginning 8 days p.i..** The peak viral load and the day the peak viral load was detected was assessed by RT-qPCR of LASV vRNA isolated from whole blood **(a)** or plaque titration of infectious virus from plasma **(b)**. For statistical comparison, the in-study control (C-4) was grouped with the three untreated LASV-challenged animals from the initial model study (**Figure 1**, n = 3) for a total n = 4. The horizontal dashed line in the leftmost panels of (a) and (b) denotes the LOQ for the assay. Statistical significance was determined using the non-parametric Mann-Whitney U-test. ns = not significant.

**b**

**a**

Arevirumab-3 (n = 5)

Tx-6/C-1

Tx-8/C-3

Tx-10/C-5

Tx-9/C-4

Controls (n = 5)

Tx-7/C-2

**Supplementary Figure 4:** **Comparison of peak viral load and day of detection in LASV-challenged macaques treated with Arevirumab-3 beginning 7 days p.i..** The peak viral load and the day the peak viral load was detected was assessed by RT-qPCR of LASV vRNA isolated from whole blood **(a)** or plaque titration of infectious virus from plasma **(b)**. For statistical comparison, the in-study control (C-5) was grouped with the three untreated LASV-challenged animals from the initial model study (**Figure 1**, n = 3) and the in-study control from the first Arevirumab-3 treatment study (**Figure 2**, n = 1) for a total n = 5. The horizontal dashed line in the leftmost panels of (a) and (b) denotes the LOQ for the assay. Statistical significance was determined using the non-parametric Mann-Whitney U-test. ** p ≤ 0.01.

**
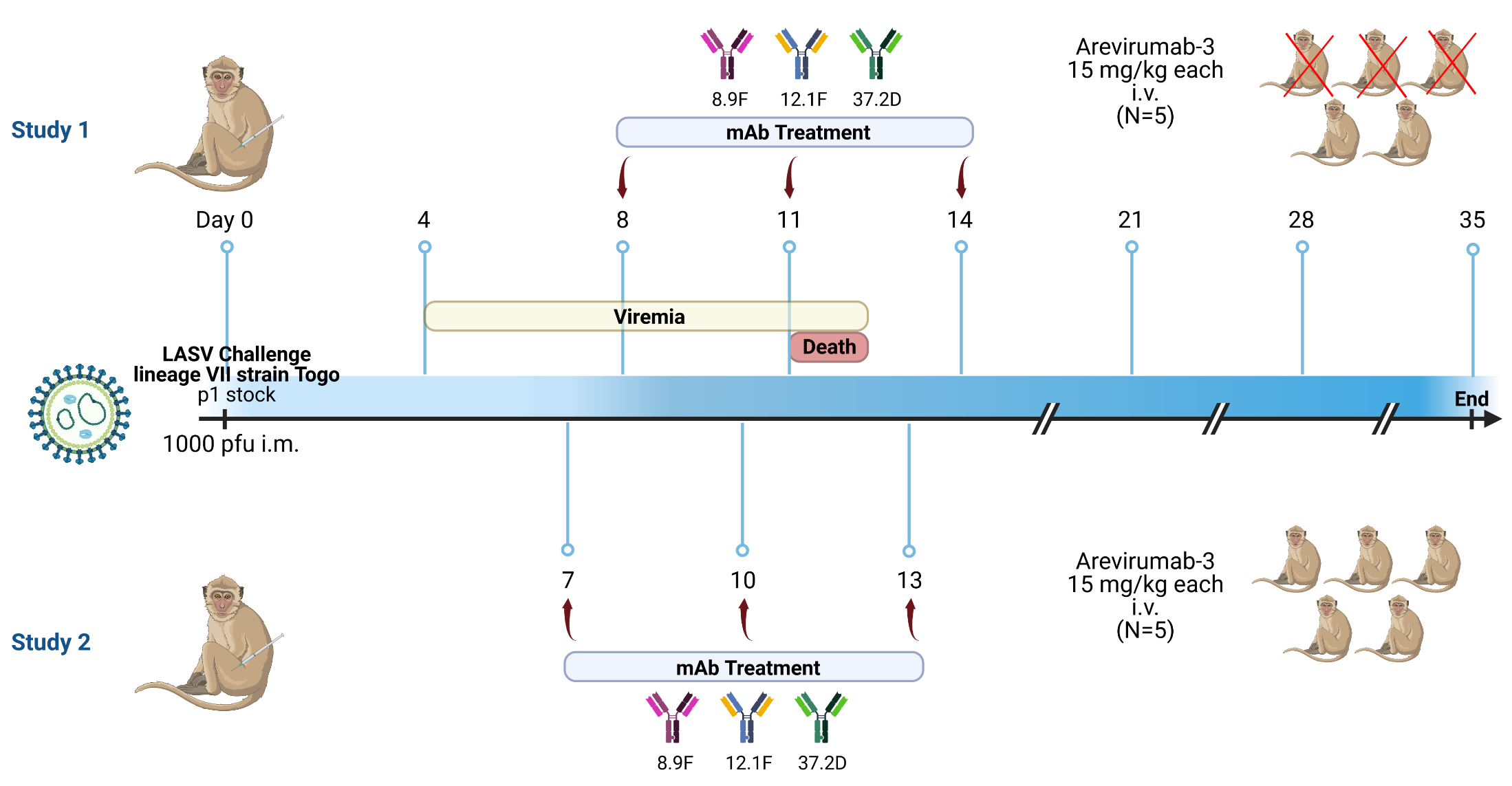
**

**Supplementary Figure 5: Schematic of experimental design for Arevirumab-3 treatment studies.** Animals were challenged with 1000 PFU of Togo by i.m. injection. Animals were treated with Arevirumab-3 on days 8, 11, and 14 after LASV challenge (Study 1) or 7, 10, and 13 after LASV challenge (Study 2). Post-exposure blood samples were collected at 4, 7 or 8, 10 or 11, 13 or 14, 21, 28, terminally, and/or 35 days.


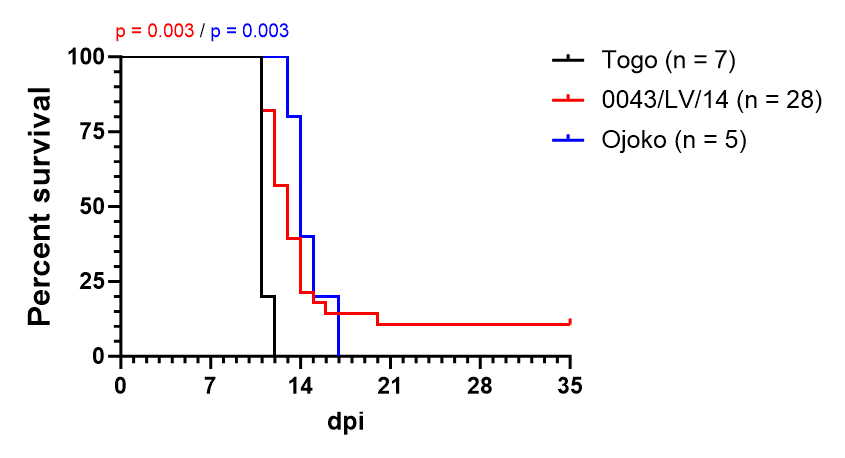


**Supplementary Figure 6:** Survival comparison of LASV Lineage VII Togo to LASV Lineage II (0043/LV/14) and Lineage III (Ojoko) isolates. The five untreated Togo-challenged positive control animals from the present study (C1 – C5) were grouped with two historical control macaques challenged with the identical LASV Togo seed stock and route (total n = 7) and compared to historical positive control LASV 0043/LV/14- (n = 28) and Ojoko-infected macaques (n = 5) [2, 3]. Statistical comparison was performed using the Mantel-Cox log-rank test and corrected for multiple comparisons using the Holm- Šídák method. Only comparisons to LASV Togo were made; differences in survival between 0043/LV/14 and Ojoko isolates were not made. Colored p-values denote statistical significance between Togo to the same colored group.

References

1. Li H, Buck T, Zandonatti M, et al. A cocktail of protective antibodies subverts the dense glycan shield of Lassa virus. Science Translational Medicine **2022**; 14:eabq0991.

2. Cross RW, Heinrich ML, Fenton KA, et al. A human monoclonal antibody combination rescues nonhuman primates from advanced disease caused by the major lineages of Lassa virus. Proceedings of the National Academy of Sciences **2023**; 120:e2304876120.

3. Cross RW, Xu R, Matassov D, et al. Quadrivalent VesiculoVax vaccine protects nonhuman primates from viral-induced hemorrhagic fever and death. The Journal of Clinical Investigation **2020**; 130:539-51.
