# Supplementary material for: Monoclonal antibody therapy demonstrates increased virulence of a lineage VII strain of Lassa virus in nonhuman primates
Source: Emerg Microbes Infect. 2024 Jan 2;13(1):2301061. doi: 10.1080/22221751.2023.2301061 (PMC10810630; doi:10.1080/22221751.2023.2301061)
Supplement: Supplementary_Methods_23November23 [file TEMI_A_2301061_SM3662.docx]

**Methods**

**Virus**

A lineage VII LASV isolate [Germany ex Togo/2016/7082](https://www.european-virus-archive.com/evag-portal?portal_search=Germany+ex+Togo%2F2016%2F7082&advanced_ictv_tax_search=Lassa+mammarenavirus) originated from serum of a LASV-infected patient in Germany that was exported from Togo [1]. The study challenge material was from the third Vero 76 cell (ATCC CRL-1587) passage of this serum. The passage two isolate was obtained from the European Virus Archive and obtained from Drs. Toni Rieger and Stephan Gunther and passed once at UTMB. The cell supernatants were stored at -80°C as ~ 1 ml aliquots. No detectable mycoplasma or endotoxin levels were measured (˂ 0.5 EU/ml).

**Antibodies**

The BNhuMAbs 8.9F, 12.1F, and 37.2D were prepared as previously described [2, 3]. Briefly, antibodies were purified by Protein A chromatography, concentrated, and formulated in a buffer for injection. Each antibody was evaluated for purity, concentration, potency, function, and identity using a panel of product release assays. Endotoxin levels were determined for each formulated antibody preparation prior to release for *in vivo* studies. *In vitro* plaque reduction neutralization tests (PRNT_50_) were performed as previously described [4].

**Nonhuman primate challenge and treatment**

Details of the study design for each experiment are provided in the Results section of the manuscript. Fifteen healthy cynomolgus macaques (*Macaca fascicularis*) of Asian origin were obtained from commercial vendors (PreLabs, Worldwide Primates, Charles River) and used to conduct three separate studies. Animals were randomized using Microsoft Excel into treatment or control groups. An initial study was performed prior to initiation of the treatment studies in order to assess the pathogenic potential of lineage VII LASV Togo. In this initial study, three adult cynomolgus macaques (C-1-C3) ~ 3.5-5 years of age and weighing 3.1-6.1 kg were exposed by intramuscular (i.m.) injection with a target dose of 1,000 PFU of LASV Togo (actual dose 1,175 PFU).

In the first therapeutic treatment study summarized in **Supplementary Figure 4** six healthy cynomolgus macaques ranging in age from ~ 5-7.5 years and weighing 2.5-3.1 kg were challenged i.m. with a target dose of 1,000 PFU of LASV Togo (actual dose 1,100 PFU). Arevirumab-3 (15 mg/kg each of MAbs 8.9F, 12.1F, and 37.2D) was administered to 5 macaques by intravenous (i.v.) infusion 8 days after LASV Togo infection. Additional i.v. doses of Arevirumab-3 were given on days 11 and 14 after LASV Togo infection. The virus positive control animal was not treated in this study. Surviving animals were euthanized at the predetermined study endpoint on day 35 after LASV Togo infection.

In the second therapeutic treatment study summarized in **Supplementary Figure 4** six healthy cynomolgus macaques ~ 2.5 years of age and weighing 2-2.9 kg were challenged i.m. with a target dose of 1,000 PFU of LASV Togo (actual dose 1,250 PFU). Arevirumab-3 (15 mg/kg each of MAbs 8.9F, 12.1F, and 37.2D) was administered to 5 macaques by intravenous (i.v.) infusion 7 days after LASV Togo infection. Additional i.v. doses of Arevirumab-3 were given on days 10 and 13 after LASV Togo infection. The virus positive control animal was not treated in this study. Surviving animals were euthanized at the predetermined study endpoint on day 35 after LASV Togo infection.

All animals for all three studies were given physical examinations, and blood was collected before virus challenge (day 0); and on days 4, 7 or 8, 10 or 11, 13 or 14, 21, 28, and 35 after virus challenge. The macaques were monitored daily and scored for disease progression with an internal LASV humane endpoint scoring sheet approved by the UTMB IACUC. UTMB facilities used in this work are accredited by the Association for Assessment and Accreditation of Laboratory Animal Care International and adhere to principles specified in the eighth edition of the Guide for the Care and Use of Laboratory Animals, National Research Council. The scoring changes measured from baseline included posture and activity level, attitude and behavior, food intake, respiration, and disease manifes­tations, such as visible rash, hemorrhage, ecchymosis, or flushed skin, and central nervous system abnormalities were scored. A score of ≥ 10 indicated that an animal met the criteria for euthanasia.

**Hematology and serum biochemistry**

Total white blood cell counts, white blood cell differentials, red blood cell counts, platelet counts, hematocrit values, total hemoglobin concentrations, mean cell volumes, mean corpuscular volumes, and mean corpuscular hemoglobin concentrations were analyzed from blood collected in tubes containing EDTA using an AcT diff laser-based hematology analyzer (Beckman Coulter, **Supplementary Tables 1-3**). Serum samples were tested for concentrations of albumin, amylase, alanine aminotransferase (ALT), aspartate aminotransferase (AST), alkaline phosphatase (ALP), blood urea nitrogen (BUN), calcium, creatinine (CRE), C-reactive protein (CRP), gamma-glutamyltransferase (GGT), glucose, total protein, and uric acid by using a Piccolo point-of-care analyzer and Biochemistry Panel Plus analyzer discs (Abaxis) (**Supplementary Tables 1-3**).

**RNA isolation from LASV-infected macaques**

On procedure days, 100 μl of blood from K2-EDTA collection tubes was collected prior to centrifugation and was added to 600 μl of AVL viral lysis buffer with 6 μL carrier RNA (Qiagen) for RNA extraction. For tissues, approximately 100 mg was stored in 1 ml RNAlater (Qiagen) for at least 24 hours for stabilization. RNAlater was completely removed, and tissues were homogenized in 600 μl RLT buffer and 1% betamercaptoethanol (Qiagen) in a 2 mL cryovial using a tissue lyser (Qiagen) and 0.2mm ceramic beads. The tissues sampled included axillary and inguinal lymph nodes, liver, spleen, kidney, adrenal gland, lung, brain, pancreas, urinary bladder, ovary or testis, uterus or prostate, conjunctiva, and eye. All blood samples were inactivated in AVL viral lysis buffer, and tissue samples were homogenized and inactivated in RLT buffer prior to removal from the BSL-4 laboratory. Subsequently, RNA was isolated from blood using the QIAamp viral RNA kit (Qiagen), and from tissues using the RNeasy minikit (Qiagen) according to the manufacturer’s instructions supplied with each kit.

**Quantification of viral load**

Primers and a probe targeting the GPC gene of LASV were used for real-time quantitative PCR (RT-qPCR) with the following primers and probe for LASV Togo: Forward: 5’- ACA GTT GCA AAT GGT GTG CT - 3’; Reverse: 5’- TGG CAG TGA TCT TCC CAT GT - 3’; Probe: 6-carboxyﬂuorescein (FAM)-5= TGC CTC TCC CAG AGT CAA GTG CA -3=-6 carboxytetramethylrhodamine (TAMRA). Viral RNA was detected using the CFX96 detection system (Bio-Rad Laboratories, Hercules, CA) in one-step probe RT-qPCR kits (Qiagen) with the following cycle conditions for LASV 0043/LV/14: 50°C for 10 min, 95°C for 10 s, and 40 cycles of 95°C for 10 s and 53°C for 30 s and the following conditions for LASV-Ojoko: 50°C for 10 min, 95°C for 10 s, and 45 cycles of 95°C for 10 s and 48°C for 30 s and Threshold cycle (CT) values representing viral genomes were analyzed with CFX Manager software, and the data are shown as genome equivalents (GEq). To create the GEq standard, RNA from viral stocks was extracted, and the number of strain-speciﬁc genomes was calculated using Avogadro’s number and the molecular weight of each viral genome.

**Plaque titration of infectious LASV**

Virus titration was performed by plaque assay using Vero 76 cells (ATCC CRL-1587) from all plasma or tissue samples as previously described [4]. Briefly, increasing 10-fold dilutions of the samples were adsorbed to Vero 76 cell monolayers in duplicate wells (200 μl) and overlaid with 0.8% agarose in 1x Eagles minimum essentials medium (MEM) with 5% FBS and 1% P/S. After 5 days incubation at 37°C/5% CO_2_, neutral red stain was added, and plaques were counted after 48-hour incubation. The limit of detection for this assay is 25 PFU/mL for plasma and 250 PFU/g for tissues.

**Histopathology and immunohistochemistry**

Tissue sections were deparaffinized and rehydrated through xylene and graded ethanols. The tissue sections were processed for IHC using the Thermo Autostainer 360 (ThermoFisher, Kalamazoo, MI). Slides were treated with Proteinase K for 5 minutes to unmask antigens (Dako, Carpenteria, CA #S3020). Sequential 15-minute incubations with avidin D and biotin solutions (Vector, Burlingame, CA #SP-2001) was performed to block endogenous biotin reactivity. Specific anti-Lassa CLD4 NP immunoreactivity was detected using an anti-LASV CLD4 NP primary antibody at a 1:1000 dilution for 60 min. Secondary antibody used was biotinylated goat anti-rabbit IgG (Vector Laboratories, Burlingame, CA #BA-1000) at 1:200 for 30 min followed by Horseradish Peroxidase Streptavidin, R.T.U (Vector Laboratories #SA-5704) for 30 min. Slides were developed with Dako DAB chromogen (Dako, Carpenteria, CA #K3468) for 5 min and counterstained with hematoxylin for 45 seconds.

**NanoString sample preparation**

We conducted targeted transcriptomics on macaque blood samples following established protocols [5]. NHPV2_Immunology reporter and capture probesets (NanoString Technologies) were hybridized with 3 µl of RNA from each sample and incubated at approximately 65°C for approximately 24 hours. The resulting RNA:probeset complexes were then loaded onto an nCounter microfluidics cartridge and processed using a NanoString nCounter SPRINT Profiler.

**Transcriptional analysis**

The nCounter .RCC files were imported into NanoString nSolver 4.0 software. To address variations in RNA inputs and reaction efficiency, we utilized a set of 10 housekeeping genes and introduced spiked-in positive and negative controls for raw read count normalization, as previously outlined [6, 7]. The NanoString nSolver Advanced Analysis module automatically selected the default array and housekeeping mRNAs. Considering that both sample input and reaction efficiency have a uniform impact on all probes, we normalized for both run-to-run and sample-to-sample variability by dividing counts within a lane by the geometric mean of the reference/normalizer probes from the same lane. The most suitable normalization genes were determined using the geNorm algorithm, as implemented in the Bioconductor package NormqPCR [8], based on the criterion of minimizing pairwise variation. Subsequently, NanoString nSolver Advanced Analysis 2.0 package was employed for differential expression analysis, generating the differential expression results and cell-type trend plots. Two samples were excluded from analysis (10 p.i. C-2 and Tx-6); all other samples met the imaging, binding, positive control, and limit of detection QC criteria. Human annotations were incorporated for each respective mRNA to enable immune cell profiling within nSolver. The resulting normalized data, including log2 ratio fold-change values and Benjamini–Hochberg adjusted p-values, were exported as an .xlsx file and imported into GraphPad Prism version 9.3.1 to create the transcript heatmap. Supplementary material containing this data is available (**Data S1**). In the heatmap analysis, we compared samples from Treated Fatal (n = 3; Tx-1,Tx-3, Tx-5) and Treated Survivor (n = 7; Tx-2, Tx-4, Tx-6, Tx-7, Tx-8, Tx-9, Tx-10) groups versus the Control cohort (n = 5; C-1, C-2, C-3, C-4, C-5) at each of the following collection timepoints: 4, 7/8, and 10/11 days p.i.. DE transcripts (Benjamini-Hochberg adjusted p-value < 0.05) were shared between the survivor groups from each study. The cell-type trend plots encompassed all analyzed samples at 10/11 days p.i.. The Venn diagram depicting overlapping DE transcripts at 10/11 days p.i. (Benjamini-Hochberg adjusted p-value < 0.05) for all three groups versus a 0 days p.i. pre-challenge baseline (**Data S1**) was produced using the online InteractiVenn tool: http://www.interactivenn.net/ [9].

References

1. Whitmer SLM, Strecker T, Cadar D, et al. New Lineage of Lassa Virus, Togo, 2016. Emerg Infect Dis **2018**; 24:599-602.

2. Mire CE, Cross RW, Geisbert JB, et al. Human-monoclonal-antibody therapy protects nonhuman primates against advanced Lassa fever. Nat Med **2017**; 23:1146-9.

3. Cross RW, Heinrich ML, Fenton KA, et al. A human monoclonal antibody combination rescues nonhuman primates from advanced disease caused by the major lineages of Lassa virus. Proceedings of the National Academy of Sciences **2023**; 120:e2304876120.

4. Geisbert TW, Jones S, Fritz EA, et al. Development of a new vaccine for the prevention of Lassa fever. PLoS Med **2005**; 2:e183.

5. Prasad AN, Woolsey C, Geisbert JB, et al. Resistance of Cynomolgus Monkeys to Nipah and Hendra Virus Disease Is Associated With Cell-Mediated and Humoral Immunity. The Journal of Infectious Diseases **2019**; 221:S436-S47.

6. Woolsey C, Cross RW, Agans KN, et al. A highly attenuated Vesiculovax vaccine rapidly protects nonhuman primates against lethal Marburg virus challenge. PLoS neglected tropical diseases **2022**; 16:e0010433.

7. Woolsey C, Fears AC, Borisevich V, et al. Natural history of Sudan ebolavirus infection in rhesus and cynomolgus macaques. Emerg Microbes Infect **2022**; 11:1635-46.

8. Vandesompele J, De Preter K, Pattyn F, et al. Accurate normalization of real-time quantitative RT-PCR data by geometric averaging of multiple internal control genes. Genome Biol **2002**; 3:RESEARCH0034.

9. Heberle H, Meirelles GV, da Silva FR, Telles GP, Minghim R. InteractiVenn: a web-based tool for the analysis of sets through Venn diagrams. BMC Bioinformatics **2015**; 16:169.
