# Supplementary material for: Monoclonal antibody therapy demonstrates increased virulence of a lineage VII strain of Lassa virus in nonhuman primates
Source: Emerg Microbes Infect. 2024 Jan 2;13(1):2301061. doi: 10.1080/22221751.2023.2301061 (PMC10810630; doi:10.1080/22221751.2023.2301061)
Supplement: Supplementary_Tables [file TEMI_A_2301061_SM3658.docx]

**Supplementary Table 1. Clinical description and outcome of Lassa Togo-challenged NHPs**

| **Subject No.** | **Sex** | **Clinical illness** | **Clinical pathology** |
| --- | --- | --- | --- |
| C-1 | M | Fever (d7,10); decreased appetite (d8-10); anorexia (d11); severe lethargy (d11); hunched posture (d11); weakness (d12); recumbency (d11); mild petechial rash (d11); seizures (d11); succumbed in PM on d11 | Thrombocytopenia (d7,10,11); monocytosis (d11); neutropenia (d7); eosinopenia (d7,10,11); basopenia (d7,10); hypoalbuminemia (d10,11); hypoproteinemia (d11); hypoamylasemia (d11); AST ↑ (d10,11); CRP ↑ (d4,7); CRP ↑↑↑↑ (d10,11) |
| C-2 | M | Decreased appetite (d6-8); anorexia (d9-12); lethargy (d11); severe lethargy (d12); hunched posture (d11,12); weakness (d11); myoclonus (d12); succumbed in PM on d12 | Leukopenia (d7,12); lymphopenia (d4,7,12); thrombocytopenia (d10); monocytopenia (d7); monocytosis (d10); neutropenia (d7,12); eosinopenia (d7,12); basopenia (d7,10,12); anemia (d12); hypoalbuminemia (d10,11); ALT ↑↑ (d10); ALT ↑↑↑↑ (d12); AST ↑ (d7); AST ↑↑ (d10); AST ↑↑↑ (d12); CRP ↑ (d7,10); CRP ↑↑ (d12) |
| C-3 | M | Fever (d10); hypothermia (d11); decreased appetite (d6); anorexia (d7-11); severe lethargy (d11); hunched posture (d11); weakness (d11); recumbency (d11); dyspnea (d11); ataxia (d11); succumbed in PM on d11 | Leukopenia (d7); lymphopenia (d4,7); thrombocytopenia (d4,7,10,11); monocytosis (d4); monocytopenia (d7); neutropenia (d7); neutrophilia (d11); eosinopenia (d4,7,10,11); basopenia (d4,7,10,11); hypoalbuminemia (d10,11); hypoproteinemia (d12); hypoamylasemia (d4,7,10,11); hyperglycemia (d11); ALT ↑ (d7); ALT ↑↑ (d10); ALT ↑↑↑↑ (d11); AST ↑ (d7); AST ↑↑↑↑ (d10,11); ALP ↑ (d11); GGT ↑ (d11); CRP ↑ (d4,7); CRP ↑↑↑ (d10); CRP ↑↑↑↑ (d11) |
|  |  |  |  |

Days after Lassa challenge are in parentheses. All reported findings are in comparison to baseline (d0) values. Decreased appetite is defined as ≤ 65% of food consumed from the previous day. Anorexia is defined as no food consumed from the previous day. Fever is defined as a temperature more than 2.5 °F over baseline, or at least 1.5 °F over baseline and ≥ 103.5 °F. Hypothermia is defined as a temperature ≤ 3.5°F below baseline. Leukopenia, lymphopenia, monocytopenia, erythrocytopenia, thrombocytopenia, neutropenia, eosinopenia, and basopenia are defined by a ≥ 35% drop in numbers of white blood cells, lymphocytes, monocytes, erythrocytes, platelets, neutrophils, eosinophils, or basophils respectively. Leukocytosis, lymphocytosis, monocytosis, neutrophilia, eosinophilia, and basophilia are defined by a 100% or greater increase in numbers of white blood cells, lymphocytes, monocytes, neutrophils, eosinophils, or basophils respectively. Anemia is defined as a concurrent ≥ 30% decrease in erythrocyte count, Hct, and Hgb. Hyperglycemia is defined as a 100% or greater increase in levels of glucose. Hypoglycemia is defined by a ≥ 25% decrease in levels of glucose. Hypoalbuminemia is defined by a ≥ 25% decrease in levels of albumin. Hypoproteinemia is defined by a ≥25% decrease in levels of total protein. Hyperamylasemia is defined as a 100% or greater increase in levels of amylase. Hypoamylasemia is defined by a ≥ 25% decrease in levels of serum amylase. Hypocalcemia is defined by a ≥ 25% decrease in levels of serum calcium. Increases in ALT, AST, ALP, CRE, CRP, Hct, and Hgb were graded on the following scale: ↑ = 2-5 fold, ↑↑ = > 5-10 fold, ↑↑↑ = > 10-20 fold, ↑↑↑↑ = > 20-fold, ↓ = ≥ 50% decrease. (BUN) blood urea nitrogen, (ALT) alanine aminotransferase, (AST) aspartate aminotransferase, (ALP) alkaline phosphatase, (CRE) Creatinine, (CRP) C-reactive protein, (Hct) hematocrit, (Hgb) hemoglobin. (LASV) Lassa virus; (M) male; (F) female.

**Supplementary Table 2. Clinical description and outcome of Lassa Togo-challenged and Arevirumab-3 treated NHPs**

| **Subject No.** | **Sex** | **Treatment** | **Clinical illness** | **Clinical pathology** |
| --- | --- | --- | --- | --- |
| Tx-1  27574 | F | Arevirumab-3  (8,11,14) | Hypothermia (d14); decreased appetite (d5-8,10-12); anorexia (d9,14); lethargy (d11-13); severe lethargy (d14); weakness (d11-14); recumbency (d14); dyspnea (d14); intention tremors (d11,12); ataxia (d13,14); succumbed in PM on d14 | Leukocytosis (d4,14); lymphopenia (d8); lymphocytosis (d11,14); thrombocytopenia (d11,14); monocytopenia (d8); monocytosis (d4,11,14); neutrophilia (d4); neutropenia (d11); eosinopenia (d8,11,14); basophilia (d4); basopenia (d8,11,14); hypoalbuminemia (d11,14); hypoamylasemia (d4,8,11); ALT ↑ (d8); ALT ↑↑↑ (d11); ALT ↑↑ (d14); AST ↑↑ (d8,11); AST ↑ (d14); ALP ↑ (d8); ALP ↑↑ (d11,14); GGT ↑ (d11); CRP ↑↑↑↑ (d4,8); CRP ↑↑ (d11); CRP ↑ (d14) |
| Tx-2  34124 | F | Arevirumab-3  (8,11,14) | Decreased appetite (d5,6,9,10) | Leukocytosis (d4,14,28); lymphocytosis (d11,14,21); thrombocytopenia (d8,11,28); monocytosis (d4,8,11,14,21,28); neutrophilia (d4,28); eosinophilia (d4,28); basophilia (d4,14,28); hypoalbuminemia (d8,11); hypoamylasemia (d4); hypoglycemia (d11,14); ALT ↑↑ (d11); ALT ↑ (d14); AST ↑ (d8,11); ALP ↑ (d11,14); CRP ↑↑↑ (d4); CRP ↑↑ (d8,28); CRP ↑ (d21) |
| Tx-3  170906 | F | Arevirumab-3  (8,11,14) | Decreased appetite (d5-11); ataxia (d11); seizures (d11); succumbed in AM on d11 | Leukopenia (d8); lymphopenia (d4,8); lymphocytosis (d11); thrombocytopenia (d8,11); monocytopenia (d8); monocytosis (d11); neutropenia (d8); eosinopenia (d8); basopenia (d8,11); hypoalbuminemia (d8,11); hypoproteinemia (d11); hypoamylasemia (d11); ALT ↑↑↑ (d11); AST ↑ (d8); AST ↑↑↑ (d11); AST ↑ (d8); ALP ↑ (d11); GGT ↑ (d11); CRP ↑ (d4,8); CRP ↑↑ (d11) |
| Tx-4  1508558 | F | Arevirumab-3  (8,11,14) | Fever (d4,8); decreased appetite (d6-8,10,12); anorexia (d9) | Leukocytosis (d14,21); leukopenia (d8); lymphopenia (d4,8); lymphocytosis (d14,21,28); thrombocytopenia (d8,11); monocytopenia (d8); monocytosis (d11,14,21); neutrophilia (d14); neutropenia (d8); eosinophilia (d4); eosinopenia (d8,35); basopenia (d8,11); hypoalbuminemia (d11); hypoglycemia (d4,8,11,14,21,35); ALT ↑ (d8,14); ALT ↑↑ (d11); AST ↑ (d8,11); ALP ↑ (d8,11,14,21); CRP ↑↑↑ (d4); CRP ↑ (d8,11,21,28); CRP ↑↑ (d35) |
| Tx-5  1603666 | F | Arevirumab-3  (8,11,14) | Hypothermia (d8,10); decreased appetite (d5-8); anorexia (d9,10); severe lethargy (d10); weakness (d10); recumbency (d10); mild petechial rash (d10); intension tremors (d10); ataxia (d10); succumbed in AM on d10 | Lymphopenia (d4,8); lymphocytosis (d10); thrombocytopenia (d8,10); monocytopenia (d4,8,10); eosinophilia (d10); basopenia (d8,10); hypoalbuminemia (d10); hypoamylasemia (d10); ALT ↑ (d8); ALT ↑↑↑↑ (d10); AST ↑↑ (d8); AST ↑↑↑↑ (d10); ALP ↑ (d10); GGT ↑ (d10); CRP ↑↑↑ (d4,8); CRP ↑↑ (d10) |
|  |  |  |  |  |
| C-4 | F | None | Hypothermia (d11); decreased appetite (d7,8); anorexia (d9-11); severe lethargy (d11); weakness (d11); recumbency (d11); mild petechial rash (d11); myoclonus (d11); succumbed in AM on d11 | Leukocytosis (d11); leukopenia (d8); lymphopenia (d8); lymphocytosis (d11); thrombocytopenia (d8,11); monocytopenia (d8); monocytosis (d4,11); neutrophilia (d11); neutropenia (d8); eosinopenia (d8); basopenia (d8); hypoalbuminemia (d8,11); hypoamylasemia (d4); BUN ↑ (d10); CRE ↑ (d10); ALT ↑↑↑↑ (d11); AST ↑ (d8); AST ↑↑↑↑ (d10); ALP ↑↑ (d10); GGT ↑↑ (d10); CRP ↑↑ (d4,8,10) |
|  |  |  |  |  |

Days after Lassa challenge are in parentheses. All reported findings are in comparison to baseline (d0) values. Decreased appetite is defined as ≤ 65% of food consumed from the previous day. Anorexia is defined as no food consumed from the previous day. Fever is defined as a temperature more than 2.5 °F over baseline, or at least 1.5 °F over baseline and ≥ 103.5 °F. Hypothermia is defined as a temperature ≤ 3.5°F below baseline. Leukopenia, lymphopenia, monocytopenia, erythrocytopenia, thrombocytopenia, neutropenia, eosinopenia, and basopenia are defined by a ≥ 35% drop in numbers of white blood cells, lymphocytes, monocytes, erythrocytes, platelets, neutrophils, eosinophils, or basophils respectively. Leukocytosis, lymphocytosis, monocytosis, neutrophilia, eosinophilia, and basophilia are defined by a 100% or greater increase in numbers of white blood cells, lymphocytes, monocytes, neutrophils, eosinophils, or basophils respectively. Anemia is defined as a concurrent ≥ 30% decrease in erythrocyte count, Hct, and Hgb. Hyperglycemia is defined as a 100% or greater increase in levels of glucose. Hypoglycemia is defined by a ≥ 25% decrease in levels of glucose. Hypoalbuminemia is defined by a ≥ 25% decrease in levels of albumin. Hypoproteinemia is defined by a ≥25% decrease in levels of total protein. Hyperamylasemia is defined as a 100% or greater increase in levels of amylase. Hypoamylasemia is defined by a ≥ 25% decrease in levels of serum amylase. Hypocalcemia is defined by a ≥ 25% decrease in levels of serum calcium. Increases in ALT, AST, ALP, CRE, CRP, Hct, and Hgb were graded on the following scale: ↑ = 2-5 fold, ↑↑ = > 5-10 fold, ↑↑↑ = > 10-20 fold, ↑↑↑↑ = > 20-fold, ↓ = ≥ 50% decrease. (BUN) blood urea nitrogen, (ALT) alanine aminotransferase, (AST) aspartate aminotransferase, (ALP) alkaline phosphatase, (CRE) Creatinine, (CRP) C-reactive protein, (Hct) hematocrit, (Hgb) hemoglobin. (LASV) Lassa virus; (M) male; (F) female.**Supplementary Table 3. Clinical description and outcome of Lassa Togo-challenged and Arevirumab-3 treated NHPs**

| **Subject No.** | **Sex** | **Treatment** | **Clinical illness** | **Clinical pathology** |
| --- | --- | --- | --- | --- |
| Tx-6  5006 | F | Arevirumab-3  (7,10,13) | Fever (d4); decreased appetite (d6,10,11); anorexia (d9) | Leukopenia (d7); lymphopenia (d4,7); thrombocytopenia (d7,10,28); monocytopenia (d7); neutropenia (d7); eosinopenia (d7); eosinophilia (d21,28); basopenia (d7,10); hypoalbuminemia (d7,10); hypoamylasemia (d35); AST ↑ (d7) |
| Tx-7  5011 | M | Arevirumab-3  (7,10,13) | Decreased appetite (d6-9,12) | Leukopenia (d4,7); lymphopenia (d4,7,10); thrombocytopenia (d7,10); monocytosis (d4,21,35); neutropenia (d4,7); neutrophilia (d13); eosinophilia (d13,21,35); basopenia (d4,7,10); hypoalbuminemia (d28,35); ALT ↑ (d10); AST ↑ (d7); CRP ↑ (d13,21,28,35) |
| Tx-8  5302 | M | Arevirumab-3  (7,10,13) | Decreased appetite (d7-9) | Leukopenia (d7); lymphopenia (d4,7); thrombocytopenia (d4,7); monocytosis (d4,10,13,21); neutropenia (d7); eosinopenia (d13); eosinophilia (d21,28); basopenia (d7,10,13); hypoalbuminemia (d7,10); AST ↑ (d7); CRP ↑ (d4,7) |
| Tx-9  5422 | M | Arevirumab-3  (7,10,13) | Decreased appetite (d5,6,8,10.11); anorexia (d7,9) | Leukopenia (d7); lymphopenia (d4,7); thrombocytopenia (d7,10,28); monocytopenia (d4,7,28,35); monocytosis (d10,13,21); neutropenia (d7,10,13,21,35); neutrophilia (d4,28); eosinopenia (d4,7,10,13,35); basopenia (d7,10); AST ↑ (d7); CRP ↑ (d4); CRP ↑↑ (d7) |
| Tx-10  5574 | M | Arevirumab-3  (7,10,13) | Fever (d4); decreased appetite (d6-8,10,12); anorexia (d9) | Leukopenia (d4,7); lymphopenia (d4,7); thrombocytopenia (d4,7,10); monocytopenia (d4); monocytosis (d13,21); neutropenia (d7,10,13,28,35); eosinopenia (d4,7,10,13); eosinophilia (d28); basopenia (d4,7,10); AST ↑ (d7); CRP ↑↑ (d4) |
|  |  |  |  |  |
| C-5 | M | None | Hypothermia (d11); decreased appetite (d5); anorexia (d6-11); lethargy (d9,10); severe lethargy (d11); weakness (d10,11); hunched posture (d9-11); recumbency (d11); ataxia (d11); paresis (d11); succumbed in PM on d11 | Leukopenia (d4,7,10,11); lymphopenia (d4,7,10,11); thrombocytopenia (d4,7,10,11); monocytopenia (d4,7,10,11); neutropenia (d7,10,11); eosinopenia (d4,7,10,11); basopenia (d4,7,10,11); hypoalbuminemia (d10,11); hypoproteinemia (d11); hyperamylasemia (d10,11); BUN ↑ (d11); ALT ↑ (d7); ALT ↑↑↑ (d10,11); AST ↑↑ (d7); AST ↑↑↑ (d10,11); CRP ↑ (d4,10); CRP ↑↑ (d7); CRP ↑↑ (d11) |
|  |  |  |  |  |

Days after Lassa challenge are in parentheses. All reported findings are in comparison to baseline (d0) values. Decreased appetite is defined as ≤ 65% of food consumed from the previous day. Anorexia is defined as no food consumed from the previous day. Fever is defined as a temperature more than 2.5 °F over baseline, or at least 1.5 °F over baseline and ≥ 103.5 °F. Hypothermia is defined as a temperature ≤ 3.5°F below baseline. Leukopenia, lymphopenia, monocytopenia, erythrocytopenia, thrombocytopenia, neutropenia, eosinopenia, and basopenia are defined by a ≥ 35% drop in numbers of white blood cells, lymphocytes, monocytes, erythrocytes, platelets, neutrophils, eosinophils, or basophils respectively. Leukocytosis, lymphocytosis, monocytosis, neutrophilia, eosinophilia, and basophilia are defined by a 100% or greater increase in numbers of white blood cells, lymphocytes, monocytes, neutrophils, eosinophils, or basophils respectively. Anemia is defined as a concurrent ≥ 30% decrease in erythrocyte count, Hct, and Hgb. Hyperglycemia is defined as a 100% or greater increase in levels of glucose. Hypoglycemia is defined by a ≥ 25% decrease in levels of glucose. Hypoalbuminemia is defined by a ≥ 25% decrease in levels of albumin. Hypoproteinemia is defined by a ≥25% decrease in levels of total protein. Hyperamylasemia is defined as a 100% or greater increase in levels of amylase. Hypoamylasemia is defined by a ≥ 25% decrease in levels of serum amylase. Hypocalcemia is defined by a ≥ 25% decrease in levels of serum calcium. Increases in ALT, AST, ALP, CRE, CRP, Hct, and Hgb were graded on the following scale: ↑ = 2-5 fold, ↑↑ = > 5-10 fold, ↑↑↑ = > 10-20 fold, ↑↑↑↑ = > 20-fold, ↓ = ≥ 50% decrease. (BUN) blood urea nitrogen, (ALT) alanine aminotransferase, (AST) aspartate aminotransferase, (ALP) alkaline phosphatase, (CRE) Creatinine, (CRP) C-reactive protein, (Hct) hematocrit, (Hgb) hemoglobin. (LASV) Lassa virus; (M) male; (F) female.

**Supplementary Table 4. H/E and IHC Severity Scores**

| **Animal ID** | **C-3** | **C-1** | **C-2** | **C-4** | **Tx-5** | **Tx-3** | **Tx-2** | **Tx-4** | **Tx-2** | **C-5** | **Tx-6** | **Tx-8** | **Tx-7** | **Tx-9** | **Tx-10** |
| --- | --- | --- | --- | --- | --- | --- | --- | --- | --- | --- | --- | --- | --- | --- | --- |
| **Treatment initiated** | NONE | NONE | NONE | NONE | Dy 8 | Dy 8 | Dy 8 | Dy 8 | Dy 8 | NONE | Dy 7 | Dy 7 | Dy 7 | Dy 7 | Dy 7 |
| Days post infection | 11 | 11 | 12 | 11 | 10 | 11 | 14 | 35 | 35 | 11 | 35 | 35 | 35 | 35 | 35 |
| Liver | 3 3 | 2 3 | 3 3 | 3 3 | 3 2 | 2 2 | 2 1 | 0 0 | 0 0 | 3 3 | 0 0 | 0 0 | 0 0 | 0 0 | 0 0 |
| Spleen | 2 3 | 2 3 | 1 3 | 2 3 | 2 2 | 2 2 | 2 1 | 0 0 | 0 0 | 2 3 | 0 0 | 0 0 | 0 0 | 0 0 | 0 0 |
| Kidney | 2 3 | 1 2 | 2 3 | 2 2 | 2 2 | 2 3 | 2 3 | 2 1 | 3 2 | 2 2 | 1 1 | 2 3 | 2 1 | 1 0 | 1 1 |
| Adrenal gland | 2 3 | 1 3 | 1 3 | 3 2 | 2 2 | 1 2 | 1 1 | 0 0 | 0 0 | 1 3 | 0 0 | 0 0 | 0 0 | 0 0 | 0 0 |
| Lung | 3 3 | 2 2 | 2 3 | 2 2 | 2 2 | 2 1 | 1 1 | 0 0 | 0 0 | 3 3 | 0 0 | 0 0 | 0 0 | 0 0 | 0 0 |
| Brain (frontal) | 2 2 | 2 2 | 3 3 | 1 1 | 2 2 | 2 2 | 1 1 | 1 0 | 1 1 | 2 2 | 1 0 | 0 0 | 1 1 | 0 0 | 0 0 |
| Brainstem | 2 3 | 2 3 | 3 3 | 2 3 | 2 3 | 3 3 | 2 2 | 2 0 | 1 1 | 2 3 | 1 0 | 1 1 | 2 1 | 2 0 | 0 0 |
| Brain (hippocampus) | 2 3 | 2 2 | 3 3 | 2 3 | 2 3 | 3 3 | 2 2 | 1 1 | 1 1 | 2 3 | 1 1 | 1 1 | 1 1 | 1 0 | 1 0 |
| Cervical Spinal Cord | 1 1 | 1 1 | 1 1 | 1 1 | 1 1 | 2 2 | 1 1 | 0 0 | 1 1 | 1 1 | 0 0 | 0 0 | 2 0 | 1 0 | 0 0 |
| Pituitary Gland | 1 1 | 1 1 | 1 1 | 1 1 | 1 1 | 1 1 | 1 1 | 0 0 | 0 0 | 1 2 | 0 0 | 0 0 | 0 0 | 0 0 | 0 0 |
| Gonad | 3 3 | 3 3 | 3 3 | 1 2 | 1 2 | 1 2 | 0 1 | 0 0 | 0 0 | 2 2 | 0 0 | 0 0 | 3 1 | 1 0 | 0 0 |
| Uterus/Prostate | 1 2 | 1 1 | 1 2 | 2 2 | 1 2 | 1 1 | 0 1 | 0 0 | 0 0 | 2 2 | 0 0 | 0 0 | 0 0 | 0 0 | 0 0 |
| Eye | 1 1 | 1 1 | 1 2 | 0 0 | 0 0 | 0 0 | 0 0 | 0 0 | 0 0 | 1 2 | 0 0 | 0 0 | 0 0 | 0 0 | 0 0 |
| GENDER | M | M | M | F | F | F | F | F | F | M | F | M | M | M | M |

| First score indicates severity score of H/E slide Second score indicates severity score of IHC slide 0 = No lesions 1 = 1-25% of examined tissues with lesions or IHC labeling, 1 cell type that is IHC positive 2 = 26-50% of examined tissues with lesions or IHC labeling, up to 2 cell types that are IHC positive 3 = 51-75% of examined tissues with lesions or IHC labeling, up to 3 cell types that are IHC positive 4 = 76-100% of examined tissues with lesions or IHC labeling, more than 3 cell types that are IHC positive |  |
| --- | --- |
|  |  |
|  |  |
|  |  |
|  |  |
|  |  |
|  |  |
|  |  |
